# Analysis of expressed sequence tags from *Actinidia*: applications of a cross species EST database for gene discovery in the areas of flavor, health, color and ripening

**DOI:** 10.1186/1471-2164-9-351

**Published:** 2008-07-27

**Authors:** Ross N Crowhurst, Andrew P Gleave, Elspeth A MacRae, Charles Ampomah-Dwamena, Ross G Atkinson, Lesley L Beuning, Sean M Bulley, David Chagne, Ken B Marsh, Adam J Matich, Mirco Montefiori, Richard D Newcomb, Robert J Schaffer, Björn Usadel, Andrew C Allan, Helen L Boldingh, Judith H Bowen, Marcus W Davy, Rheinhart Eckloff, A Ross Ferguson, Lena G Fraser, Emma Gera, Roger P Hellens, Bart J Janssen, Karin Klages, Kim R Lo, Robin M MacDiarmid, Bhawana Nain, Mark A McNeilage, Maysoon Rassam, Annette C Richardson, Erik HA Rikkerink, Gavin S Ross, Roswitha Schröder, Kimberley C Snowden, Edwige JF Souleyre, Matt D Templeton, Eric F Walton, Daisy Wang, Mindy Y Wang, Yanming Y Wang, Marion Wood, Rongmei Wu, Yar-Khing Yauk, William A Laing

**Affiliations:** 1 The Horticultural and Food Research Institute of New Zealand, PB 92169, Auckland, New Zealand; 2 Max-Planck-Institute of Molecular Plant Physiology, Am Mühlenberg 1, 14476 Potsdam-Golm, Germany

## Abstract

**Background:**

Kiwifruit (Actinidia spp.) are a relatively new, but economically important crop grown in many different parts of the world. Commercial success is driven by the development of new cultivars with novel consumer traits including flavor, appearance, healthful components and convenience. To increase our understanding of the genetic diversity and gene-based control of these key traits in Actinidia, we have produced a collection of 132,577 expressed sequence tags (ESTs).

**Results:**

The ESTs were derived mainly from four *Actinidia *species (*A. chinensis, A. deliciosa, A. arguta *and *A. eriantha*) and fell into 41,858 non redundant clusters (18,070 tentative consensus sequences and 23,788 EST singletons). Analysis of flavor and fragrance-related gene families (acyltransferases and carboxylesterases) and pathways (terpenoid biosynthesis) is presented in comparison with a chemical analysis of the compounds present in *Actinidia *including esters, acids, alcohols and terpenes. ESTs are identified for most genes in color pathways controlling chlorophyll degradation and carotenoid biosynthesis. In the health area, data are presented on the ESTs involved in ascorbic acid and quinic acid biosynthesis showing not only that genes for many of the steps in these pathways are represented in the database, but that genes encoding some critical steps are absent. In the convenience area, genes related to different stages of fruit softening are identified.

**Conclusion:**

This large EST resource will allow researchers to undertake the tremendous challenge of understanding the molecular basis of genetic diversity in the *Actinidia *genus as well as provide an EST resource for comparative fruit genomics. The various bioinformatics analyses we have undertaken demonstrates the extent of coverage of ESTs for genes encoding different biochemical pathways in *Actinidia*.

## Background

**Figure 1:**
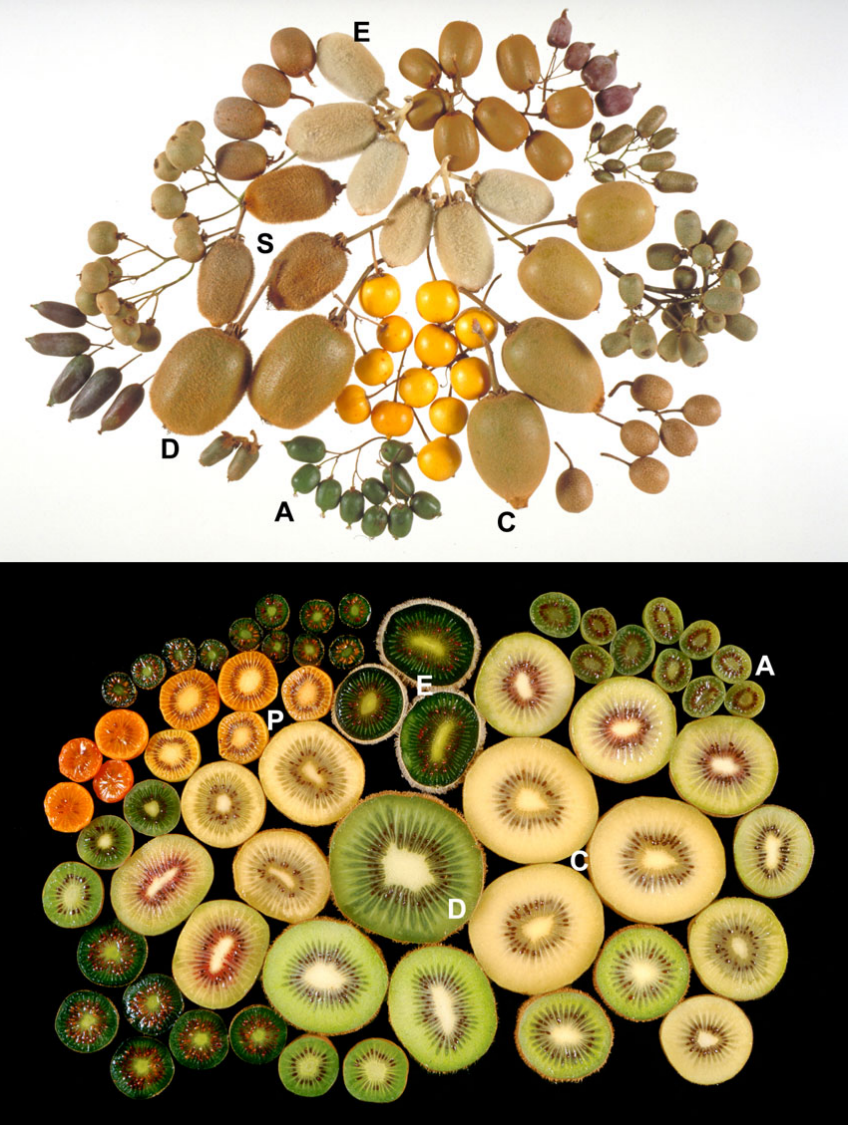
Fruit diversity in the genus *Actinidia*. Fruit of species used to make EST libraries are identified by letters. A is *A. arguta*, C is *A. chinensis*, D is *A. deliciosa*, E is *A. eriantha*, I is *A. indochinensis*, P is *A. polygama *and S is *A. setosa*. A. *hemsleyana *is not in the photos.

The genus *Actinidia *Lindl. is large, containing between 50 and 70 species of climbing plants originating mainly in southern China [1]. Over the past 30 years kiwifruit has developed into an important horticultural crop, firstly in New Zealand, and subsequently in other countries such as Chile, China and Italy [2,3]. Currently cultivars from three species are grown commercially; the green-fleshed kiwifruit, *Actinidia deliciosa *(A. Chev.) C.F.Liang et A.R.Ferguson, the closely related yellow-fleshed *A. chinensis *Planch. and the kiwiberry, *A. arguta *(Sieb. et Zucc.) Planch. ex Miq. Most of the kiwifruit cultivars grown commercially are seedling selections and there has been little systematic breeding [2]. Consequently there are still many characteristics within the genus (Fig. 1A) that could be incorporated into commercial cultivars [1] and to do this efficiently requires a better knowledge of how these characteristics are regulated. For the consumer, critical desirable attributes of kiwifruit are flavor and fragrance, appearance, healthful components and convenience.

Flavor and fragrance are determined by the chemical composition of the fruit. A wide range of compounds has been detected within the *Actinidia *genus leading to distinct and different flavors in the fruit [4]. These compounds include polyphenolics, acids, alcohols and volatile compounds such as terpenes and esters. Flavor is also influenced by the sugar to acid balance, with some growers currently receiving a premium for sweeter fruit. *Actinidia *fruit color ranges from the original green kiwifruit, newer yellow varieties as well as red, purple and orange fruit (Fig. 1B). The extensive range of colors is caused by the presence or absence of chlorophyll, anthocyanins and carotenoids [4]. Healthful attributes of kiwifruit include its high ascorbic acid levels [2], quinic acid levels [5], and the presence of triterpenes and folic acid. On the other hand, allergens are identified as undesirable health attributes in kiwifruit and in many other fruit [4]. Convenience includes such factors as the ability to store the fruit for a long time [2] and a long shelf life, as well as easy determination of eating ripeness and having an edible or peelable skin. Little is currently known about the genetic diversity and gene-based control of these major attributes in kiwifruit.

EST databases as resources for uncovering genetic diversity have been established for many plant species (Additional file 1). Most comprehensively surveyed are *Arabidopsis *(*Arabidopsis thaliana*; 1,279,945 in GenBank) and rice (*Oryza sativa*; 1,211,418 in GenBank), both of which have been fully genome-sequenced. Fruit crops have been less extensively surveyed, but recently there have been reports on EST projects from fruit, including tomato (*Lycopersicon esculentum *[6], 258,408 sequences in GenBank), grape (*Vitis vinifera*, which has also had its genome fully sequenced [7]; [8,9], 363,365 ESTs in GenBank), apple (*Malus *× *domestica *[10], 255,103 sequences in GenBank) and pineapple (*Ananas comosus *[11], 5649 ESTs in GenBank). However, there are few ESTs sequenced from the Ericales (the order to which *Actinidia *belongs) registered in GenBank (Additional file 1). These significant fruit EST resources have been used to identify genes likely to be involved in the ripening process in tomato [6] and in the generation of aroma in apple [12]. In addition, ESTs are useful sources of simple sequence repeats (SSRs) and single-nucleotide polymorphisms (SNPs), both of which are useful markers for creating genetic maps in plants [13-19].

Knowledge of the *Actinidia *genome or transcriptome is currently restricted to the 511 sequences available in GenBank (dbEST, January 2008). To improve and develop new varieties of kiwifruit with the desired flavor, health and convenience attributes, it is useful to use modern genomics techniques in conjunction with breeding tools such as marker-assisted selection and genetic transformation. For this reason, we undertook a major EST sequencing project in *Actinidia *to develop a basic genetic resource covering a range of different species and tissues. In this paper we analyze more than 130,000 ESTs, derived mainly from four species in the *Actinidia *genus (Table 1). This information is presented with reference to the chemical composition of these species and using specific examples where we have increased our understanding of the genetic diversity and gene-based control of critical attributes.

**Table 1:** Numbers of ESTs obtained from different *Actinidia *tissues by species.

	Species					
Tissue	*A. arguta*	*A. chinensis*	*A. deliciosa*	*A. eriantha*	Other^a^	Total^b^

Bud	^c^	15,689	3,4519			50,208
Cell^d^		4,851				4,851
Fruit	5,421	8,453	13,282	11,259		38,415
Leaf		17,325				17,325
Petal	1,836	1,061	9,950	1,388	1,422	15,657
Root					5,101	5,101
Stem					1,020	1,020

Total	7,257	47,379	57,751	12,647	7,543	132,577

The number of ESTs was calculated by amalgamating libraries from the same tissues.

^a ^Other miscellaneous species were *Actinidia setosa *(1,020 from stem), *A. hemsleyana *(5,101 from roots), *A. polygama *(1,348 from petal) and *A. indochinensis *(74 from petal).

^b ^See Additional file 2a for full details of libraries and tissues.

^c ^Empty cells had no ESTs sequenced.

^d ^Cell refers to cell culture.

## Results

### Overview

*Actinidia *cDNA libraries were constructed mainly from four *Actinidia *species: *A. deliciosa, A. chinensis, A. eriantha *and *A. arguta *(Table 1, Additional file 2). The libraries were constructed from petals, fruits, buds and leaves, with a small number from roots and cell culture and sequenced from the 5' end. The average edited sequence length of the 132,577 ESTs was 503 bases. Clustering these sequences using a 95% threshold resulted in 18,070 tentative consensus (TC) sequences (average length 577 bases), with 23,788 ESTs remaining as singletons. The combination of TCs and singletons are referred to as non redundant (NR) clusters. These numbers are similar to those observed with apple [10]. Over 95% of TCs had fewer than 18 EST members, and the largest single TC had 758 members. It should be noted that *A. chinensis *and *A. deliciosa *are more closely related to each other than the other species mentioned in this paper, and that only a restricted number of genotypes within each species was used in making EST libraries compared to the total numbers available.

### Sequence analysis

#### Codon usage

Knowledge of the GC content of a genome and codon usage is useful when devising PCR-based strategies for mapping and gene isolation, as well as for hybridization studies by microarray. Based on the coding regions of 302 *A. deliciosa*, 319 *A. chinensis *and 84 *A. eriantha *full-length cDNAs, the GC content in the third base position was estimated to be 55%, 49% and 58%, respectively. These values are higher than the overall GC ratio from the sequences of the complete EST datasets of *A. deliciosa *(46%), *A. chinensis *(46%) and *A. eriantha *(48%), indicating that there is some pressure, particularly in *A. deliciosa *and *A. eriantha*, towards an increased GC ratio in coding regions compared with non-translated regions. Overall the codon usage of the three *Actinidia *species is similar, although not identical (Additional file 3). *A. deliciosa *and *A. eriantha *differ in only their preference for aspartate (GAT/GAC) and serine (TCT/TCC). In contrast, comparative codon preferences between *A. deliciosa *and *A. chinensis *show differences for alanine (GCC/GCT), glycine (GGC/GGA), isoleucine (ATC/ATT), leucine (CTC/TTG), asparagine (AAC/AAT), glutamine (CAG/CAA), threonine (ACC/ACT) and valine (GTG/GTT). In most of these codons, *A. chinensis *shows a greater preference for an A or T in the third base position, accounting for its lower GC third base percentage when compared with the other two species.

#### Polymorphisms and genetic markers

Both SNPs and microsatellites or SSRs are valuable tools for genetic mapping within breeding populations of many crops. A total of 32,764 biallelic SNPs were detected from the overlapping regions of 3,901 (21.6%) of the 18,070 TCs, at a rate of one SNP every 417 bp. This frequency is higher than that reported in apple using a dataset of comparable size (one SNP per 706 bp from ~150,000 ESTs [10]). As several *Actinidia *species were used to construct the cDNA libraries, while the apple cDNA libraries were from different varieties of the same species, this increased frequency of SNPs could reflect the greater genetic diversity in the surveyed *Actinidia *EST libraries. For example the frequency in apple increased significantly to 1 in 149 bp when a greater diversity of genotypes was added (Chagne *et al *unpublished results). The polyploidy nature of several of the *Actinidia *species used may also have affected SNP frequencies. As a result of this, some of the SNPs identified may not be allelic in nature but due to homoeologous or paralogous sequences clustering in the same TC.

EST-derived SSRs have already been shown to be valuable mapping tools in *Actinidia chinensis *[14] where 150 SSRs with more than 10 dinucleotide repeat units were tested as markers in an *A. chinensis *mapping population. More than 90% of the SSR markers were polymorphic and segregated within the population. Subsequently, 20 of these SSRs were shown to be transportable across multiple species of *Actinidia*, showing the value of this resource [15].

Compared with apple [10], where less than 20% of the NRs contained a microsatellite, over 30% of *Actinidia *NRs were found to have at least one. The other major difference between apple and *Actinidia *was that, while di- and tri-nucleotide repeats were equally frequent in apple at 7 to 8% frequency, di-nucleotide repeats (18%) were twice as frequent as tri-nucleotide repeats (9%) in *Actinidia*. Tetra-nucleotide repeats were found at similar frequencies in NRs for the two genera. The lengths of the SSRs were similar in both apple and *Actinidia*, with 50% of the repeats having between 12 and 14 bases. As in apple, AG repeats were the most frequent (16% of NRs, significantly more frequent than in apple) and were followed by AT (1.2%) and AC (0.8%) while GC repeats were very infrequent.

The position of SSRs in relation to the putative initiation ATG was very similar when comparing *Actinidia *with other plants such as *Arabidopsis *and apple [10,20], with di-nucleotide repeats being predominantly (93%) in the 5' UTR, and tri-nucleotide repeats being more evenly distributed along the gene (37%).

#### Comparative genomic DNA hybridizations by microarray

**Figure 2:**
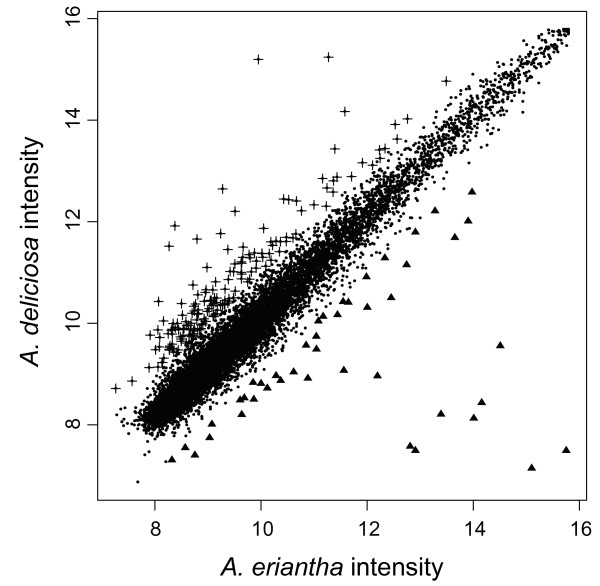
Plot of *Actinidia deliciosa *versus *A. eriantha *microarray intensities. Genomic DNA was labeled with cy3 and cy5 in two separate reactions, combined and hybridized to a single microarray slide. Spots not found or flagged "bad" were removed and each channel was normalized using a quantile normalization. Of the two arrays, 15,024 were flagged "good" using *A. deliciosa *DNA and 13,785 were flagged "good" using *A. eriantha *DNA. Of these spots, 13,443 were common to both species. Black spots represent oligonucleotides that showed similar intensity of hybridization between the two species. Triangles represent oligonucleotides that showed an increase in intensity with *A. eriantha *genomic DNA and crosses represent oligonucleotides that showed an increase using *A. deliciosa *genomic DNA.

A 17,472 feature *Actinidia *oligonucleotide microarray was constructed based on sequence data from the *Actinidia *EST database. The 44–55 mer oligonucleotides were derived from gene sequences of different *Actinidia *species (Additional file 4). To test the cross hybridization between the different genomes of the *Actinidia *species, genomic DNA from two *Actinidia *species (*A. deliciosa *and *A. eriantha*) was hybridized to two different microarray slides. A plot of average signal intensity for the two species against each other is shown in Fig. 2. Of the 13,443 informative features, the majority (98.4%) showed a similar level of hybridization between the two species (less than an average of 2-fold change), with 172 showing a higher signal intensity in *A. deliciosa *(plus symbols, Fig. 2), and 44 showing a higher intensity in *A. eriantha *(triangles, Fig. 2). There was no correlation between the species from which the oligonucleotide was derived and the increased or decreased level of hybridization (Table 2). Consequently, the increases are probably due to separate gene duplications in each species.

**Table 2:** Comparative genomic hybridizations of different *Actinidia *species.

	*A. arguta*	*A. chinensis*	*A. deliciosa*	*A. eriantha*	*A. hemsleyana*	*A. indo-chinensis*	*A. poly-gama*	*A. setosa*
No. of oligos^a^	479^b^	8,957	6,606	978	148	12	188	104
A	2	58	95	15	0	0	1	1

B	0	12	27	5	0	0	0	0

A # features where the signal is increased in *A. deliciosa*.

B Signal increased in *A. eriantha*.

^a ^The number of oligonucleotide primers on the microarray derived from each Actinidia species.

^b ^Oligonucleotide microarrays were hybridized with labeled genomic DNA from A. deliciosa or A. eriantha. The signals were normalized and selected as differentially changing between the two species using a threshold of two.

### Functional analysis

#### Highly populated tentative consensus sequences

The TC with the greatest number of ESTs was a cysteine proteinase (actinidin family, 758 members) followed by a metallothionein (547 members) (Additional file 5). However, when similar proteins were taken into account (i.e. different TCs that all matched a common *Arabidopsis *protein as their closest homolog with E < 1.0e^-100^), there were 1,266 ESTs matching cysteine proteinases followed by 769 metallothionein protein-encoding ESTs.

It was not surprising to identify the cysteine protease actinidin as the most highly abundant TC cluster in *Actinidia*. Actinidin can constitute up to 50% of soluble protein in mature *A. deliciosa *fruit at harvest [21]. Recent research has shown that this cysteine protease exists in basic and acidic forms and the amount of each form varies between species, with fruit of the important *A. chinensis *cultivar 'Hort16A' being almost devoid of acidic actinidin [22]. ESTs for actinidin occur throughout the fruit libraries that make up the database, and these cluster into 10 distinct genes/alleles [22]. As two of the key fruit libraries were subtracted for actinidin, the number of ESTs in the TC is likely to be an under-representation of the actual number. The function of this protease may be related to insect defense [23].

The metallothionein TC cluster contains a gene (pKIWI504) previously identified in kiwifruit [24] as being highly expressed in young fruit with reduced expression in the later stages of fruit development. However, the *in vivo *function of the metallothionein TC cluster is currently unknown. Interestingly, pineapple also had a high proportion of metallothionein ESTs [11].

Fourteen TCs in *Actinidia *with their proteins annotated by BiolView as "not assigned-unknown" proteins (i.e. matches to sequences with unknown function from *Arabidopsis *or other species) were highly expressed with over 100 EST members. Four of the unassigned TCs contained more than 300 EST members. These four TCs included kiwellin [25], which is a homolog to a grape ripening-related protein [26] with no *Arabidopsis *homologs (527 EST members); a homolog of At5g11420, a protein of unknown function (339 EST members); a senescence-related protein (no *Arabidopsis *matches, 311 EST members); and an ABA stress-related protein (no *Arabidopsis *matches, 302 EST members).

#### Functional analysis using Mapman and InterPro

Functional analysis of ESTs and NRs using Mapman [27] is shown in Table 3. The analysis was conducted by identifying each NR's nearest homolog to *Arabidopsis*, using BLASTx and identifying domains/families that are indicative for a given function using InterProScan. Then that NR and its member ESTs were assigned to the corresponding bin of the *Arabidopsis *match. Of the 41,858 NRs, 28,345 had sufficient homology to an *Arabidopsis *sequence to assign it a functional classification in Mapman. Thus, 32% of NRs from *Actinidia *had no *Arabidopsis *homolog, but a proportion would have homologs in other crops (e.g. genes related to fruit ripening).

**Table 3:** Functional classification of ESTs and NRs from *Actinidia*.

Bin Code	Bin Name	% *Actinidia * ESTs^a^	% *Actinidia *NR sequences^b^	% *Arabidopsis * sequences	% Potato NRs
1	PS	2.18	1.22	0.73	0.75
2	major CHO metabolism	0.86	0.72	0.37	0.54
3	minor CHO metabolism	1.02	0.75	0.45	0.41
4	glycolysis	1.03	0.60	0.23	0.27
5	fermentation	0.38	0.21	0.05	0.07
6	Gluconeogenesis/glyoxylate cycle	0.14	0.11	0.04	0.02
7	OPP	0.25	0.22	0.11	0.12
8	TCA/org. transformation	0.64	0.55	0.27	0.27
9	mitochondrial electron transport/ATP synthesis	0.82	0.71	0.49	0.34
10	cell wall	3.20	2.27	1.90	1.11
11	lipid metabolism	1.78	1.91	1.49	1.32
12	N-metabolism	0.08	0.16	0.09	0.08
13	amino acid metabolism	2.64	2.00	1.07	1.18
14	S-assimilation	0.10	0.08	0.05	0.05
15	metal handling	2.53	0.59	0.34	0.20
16	secondary metabolism	3.02	2.09	1.64	1.42
17	hormone metabolism	2.16	1.99	2.27	1.69
18	co-factor and vitamin metabolism	0.10	0.14	0.16	0.15
19	tetrapyrrole synthesis	0.28	0.26	0.17	0.16
20	stress	4.08	3.33	3.48	2.64
21	redox. regulation	1.02	1.00	0.70	0.64
22	polyamine metabolism	0.29	0.12	0.05	0.07
23	nucleotide metabolism	0.59	0.68	0.57	0.49
24	biodegradation of xenobiotics	0.04	0.08	0.09	0.06
25	C1-metabolism	0.19	0.19	0.14	0.12
26	miscellaneous	4.72	4.59	5.38	3.69
27	RNA	7.86	9.86	10.88	6.82
28	DNA	1.60	2.05	1.99	1.25
29	protein	18.61	16.94	13.31	9.59
30	signalling	3.42	4.77	4.46	3.10
31	cell	3.23	3.04	2.57	1.68
33	development	1.29	1.58	2.24	1.33
34	transport	3.62	3.99	3.49	2.58
35	not assigned	26.23	31.21	38.72	55.79

^a ^Actinidia NRs were assigned to a Mapman (Thimm et al., 2004) bin according to their nearest Arabidopsis BLAST hit. ESTs belonging to the Actinidia NR were then assigned to the same bin.

^b ^Potato and Arabidopsis functional assignments were as described [27,100]

A comparison of the percentage of NRs assigned to the top level Mapman classification bins and the percentage of *Arabidopsis *genes classified in the same bin showed a strong relationship with an r^2 ^value of 0.96 and a slope of 0.87, near to the expected 1:1 slope. The same relationship when calculated for second level bins gave an r^2 ^of 0.98 and a slope of 1.14 (second level bin data not shown). Taking only those top level bins that contained more than 100 NRs to remove chance as a major factor, only two bins appeared to be over-represented (i.e. > 2.0 times higher than the mean) in *Actinidia*, namely bin 4 (glycolysis) and bin 8 (TCA cycle). This may reflect a high level of primary metabolism in fruit. Interestingly, when all NRs are included, bin 5 (fermentation) and bin 6 (gluconeogenesis) were added to the list of over-represented NRs. The same relationships for potato showed no anomalies (Table 3). This analysis demonstrates that the clustering of ESTs generated a fair representation of the distribution of genes.

Overall, the average number of ESTs per NR in the database was 4.0. There were only two bins with a high number of ESTs per NR, bin 15 (metal handling) with over 17 ESTs per NR and bin 22 (polyamine metabolism) with over 9. However, bin 22 had a low number of NR and EST members, suggesting this result may have occurred by chance. In contrast, bin 15 included the metallothioneins and the very highly expressed EST discussed earlier (Additional file 5).

TCs with high numbers of ESTs (> 100) came from 18 of the 35 Mapman bins [27] with approximately three quarters falling into the bin names "not assigned, no ontology", "protein degradation", "metal handling binding chelation and storage", "stress biotic", "cell wall degradation" and "secondary metabolism isoprenoids".

The most common InterPro families [28] were also analyzed (Additional file 6). There were 3,111 InterPro families (flagged as true by InterPro) represented in the *Actinidia *NRs, with the most NRs found in the protein kinases grouping. Following that were thioredoxins, while cysteine proteinases, which had the highest number of ESTs in the database, were represented as the 7^th ^most frequently occurring NR.

### Genes related to flavor and fragrance

Understanding the relationship between fruit flavor and the genetic diversity present in the EST database requires a detailed analysis of the flavor and fragrance compounds present in the *Actinidia *genus. Fruit and flower samples from the major *Actinidia *varieties that were used to produce the EST database were characterized using both headspace sampling and solvent extraction followed by GC-FID/MS analysis. The results of this compound analysis are presented in Additional file 7 for esters (over 55 identified compounds), acids, alcohols and terpenoids.

#### Compound analysis

The most abundant volatile compounds in the flowers of these *Actinidia *species were alcohols and terpenes. *A. chinensis *flowers contained 71% sesquiterpenes, largely dominated by farnesols (Additional file 7). *A. deliciosa *flowers had the highest volatile ester content at around 12%, and aromatic (6–9%) and straight-chain alcohols (9%). These flowers were notable for their α-farnesene (12–26%) and germacrene D (0.3–12%) content. *A. arguta *flowers contained branched-chain (13%) and aromatic alcohols (14–50%), and monoterpenes (12–24%), which were mostly linalool oxidation products reported previously [29]. *A. eriantha *flowers contained few volatile compounds, the notable ones being 2-phenylethanol (8.5%), 6-methyl-5-hepten-2-one (also 8.5%), and β-myrcene (5%). The flowers from two further *Actinidia *species showed distinct volatile profiles. *A. polygama *flowers contained linalool oxides (36.5%) and dehydroiridodial (37%), a monoterpene related to the *Nepeta cataria *(catnip) compounds [30,31]. The flowers of *A. indochinensis *flowers were dominated by 2-phenylethanol (25.8%) and a couple of terpenes, the major one being linalool (23.6%). The aromas from these flowers have also had descriptors attached (McNeilage et al. unpublished observations). For example *A. eriantha *has been described as coconut and berry, and *A. arguta *with descriptors including sweet, lime, rose and vanilla [29]. *A chinensis *and *A. deliciosa *are described as having tea-rose aromas, sweet, fresh, lilac and violet, while *A. polygama *has gardenia and lime descriptors. These descriptors reflect the diversity of volatile compounds found in the *Actinidia *flowers.

In fruit, esters accounted for up to 85% of the total volatiles. The main *A. chinensis *esters were the fresh, fruity ethyl butanoate which, depending upon analytical method and genotype, ranged from 3 to 54%, and butyl butanoate, both of which are common fruit esters [32]. *A. chinensis *'Hort16A' fruit contained significant amounts of eucalyptol (0.2 to 21%) which has been described as having a fresh, pungent cooling taste [32]. Acetaldehyde, hexanal, E-2-hexenal and ethyl butanoate are known to be important contributors to Hort16A aromas [33]. Ethyl butanoate along with methyl butanoate and ethyl acetate were the major (~60%) compounds in the green *A. deliciosa *fruit and are known to be major volatile contributors to its flavor [34]. The other major compound was ethanol (16%), with only trace levels of other alcohols and terpenes. Whilst at more modest levels in *A. arguta *fruit (2–8%), ethyl butanoate was still substantial. *A. arguta *fruit were notable for their content of methyl- and ethyl benzoate, which are responsible for the distinctive aroma of feijoa (*Feijoa sellowiana *O. Berg) fruit [35], and also camphor (7%). The dominant ester in *A. eriantha *fruit was butyl acetate (12%), a flavor compound found in many fruit, which has a strong, fruity odor and a taste reminiscent of pineapple [32,36]. However, the total volatile level in *A. eriantha *is lower than other species and the number of esters is also lower (Additional file 7).

#### Ester biosynthesis

**Figure 3:**
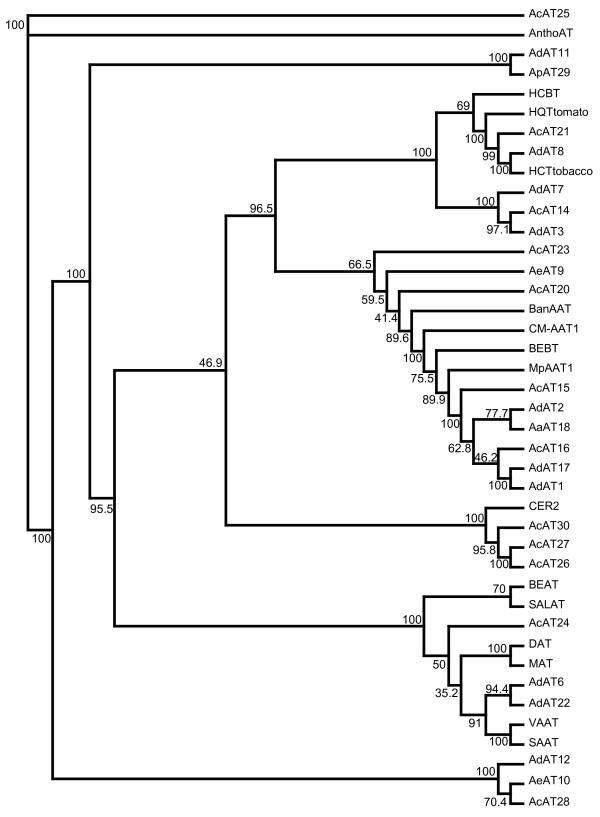
Phylogenetic tree of plant acyltransferases (ATs) of known function and *Actinidia *members of the acyltransferase family. Previously published plant acyltransferase sequences from GenBank were used to identify genes from the *Actinidia *EST database using BLAST searches. Abbreviations for species and AT names are as follows: AdAT, *Actinidia deliciosa *AT; AeAT, *A. eriantha *AT; AcAT, *A. chinensis *AT; AaAT, *A arguta *AT; DAT, *Catharanthus roseus *deacetylvindoline 4-0-acetyltransferase, GenBank Accession No. AF053307; MAT, *C. roseus *minovincinine 19-hydroxy-O-acetyltransferase, AAO13736; BEAT, *Clarkia breweri *acetyl-CoA:benzylalcohol acetyltransferase, AF043464; SALAT, *Papaver somniferum *salutaridinol 7-O-acetyltransferase, AF339913; BEBT, *Clarkia breweri *benzoyl-CoA:benzyl alcohol benzoyl transferase, AF500200; MpAAT1, *Malus pumila *alcohol acyltransferase; AY707098; CM-AAT1, *Cucumis melo *alcohol acyltransferase, CAA94432; SAAT, *Fragaria × ananassa *alcohol acyltransferase, AAG13130; HCBT, *Dianthus caryophyllus *anthranilate N-hydroxycinnamoyl benzoyltransferase, Z84383; AnthocyaninAT *Petunia frutescens *anthocyanin acyltransferase, BAA93453; VAAT, *Fragaria vesca *alcohol acyltransferase, AX025504; BanAAT, *Musa acuminata *alcohol acyltransferase, AX025506; CER2, *A. thaliana *CER2 gene, X93080; HCT, *Nicotiana tabacum *hydroxyl-cinnamoyl transferase, AJ507825; HQT, *Lycopersicon esculentum *hydroxycinnamoyl CoA quinate transferase, AJ582652. Percentage bootstrap values (1000 bootstrap replicates) for groupings are given by each branch. Transf. on cladogram is transferase.

Esters are synthesized from their acid and alcohol precursors by acyltransferases (ATs). We identified 30 ATs in the *Actinidia EST *database, of which 25 are full length. These sequences were analyzed for their phylogenetic relationship with other plant ATs of known function (Fig. 3). Two clades were identified that contained enzymes involved in the synthesis of flavor-related esters (Clades 1 and 2 in Fig. 3). The first contained alcohol acyltransferases (AATs) from apple, melon and banana and nine AATs from *Actinidia *(AdAT1, AdAT2, AeAT9, AcAT15, AcAT16, AdAT17, AaAT18, AcAT20, AcAT23), while the second clade contained AATs from strawberry and three AATs from *Actinidia *(AdAT6, AdAT22, AcAT24). An *Actinidia *AT predicted to be involved in esterifying anthocyanins (AcAT25) and two ATs predicted to be anthranilate acyltransferases (AdAT8, AcAT21) were also identified. Three *Actinidia *ATs were closely related to the carboxylesterase-related protein 2 CER2 and therefore predicted to be elongases (AcAT30, AcAT27 and AcAT26) possibly involved in wax production.

Multiple sequences of the same genes were recovered for some genes reflecting orthologs, alleles or sequences from different genomes within polyploid genomes. For example, the three ATs, AcAT16, AdAT17 and AdAT1, and the two ATs, AaAT18 and AdAT2, are probable variants of just two different AATs. *A. deliciosa *is a hexaploid (6n) possibly explaining this multiplicity of genes. We may have uncovered at least one instance where, for a single AAT, all three genomic versions have been isolated (AcAT16, AdAT17 and AdAT1).

Carboxylesterases conduct the opposite reaction to ATs, i.e. the hydrolysis of esters into acids and alcohols [37]. As well as ester hydrolysis, plant carboxylesterases have been implicated in isoflavonoid biosynthesis [38], plant defense [39], and hormone regulation [40]. Analysis of *Actinidia *flowers and fruits showed a wide range of acids and alcohols (Additional file 7).

Previously published plant carboxylesterase (CXE) genes from GenBank were used to identify CXE genes in the *Actinidia *EST database using BLAST searches. The sequence alignment of CXEs revealed amino acid motifs and secondary structural features characteristic for members of the α/β hydrolase fold superfamily and more particularly of CXEs such as an active site serine surrounded by a GXSXG motif.

**Figure 4:**
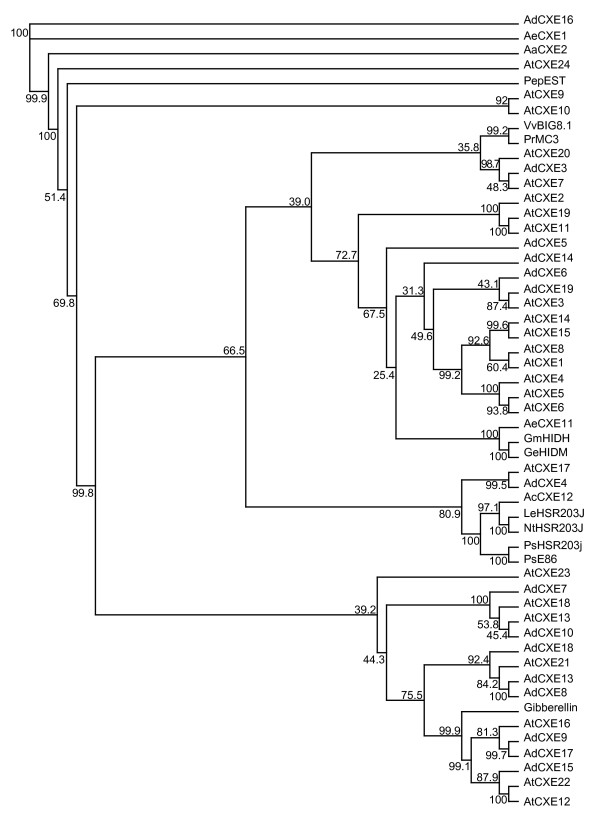
Phylogenetic tree of plant carboxylesterases of known function and *Actinidia *members of the carboxylesterase family (CXE). Previously published plant carboxylesterase sequences from GenBank were used to identify genes in the *Actinidia *EST database using BLAST searches. Abbreviations for species and CXE names are as follows: AtCXE, *Arabidopsis thaliana*, CXE; AaCXE, *A. arguta *CXE; AcCXE, *A. chinensis *CXE; AdCXE, *A. deliciosa *CXE; AeCXE, *A. eriantha *CXE; PepEST, *Capsicum annuum *Esterase, GenBank Accession No. AAF77578.1; Gibberellin, Gibberellin receptor GID1, Q6L545; LeHSR203J, *Lycopersicon esculentum *HSR203J, BAA74434.1; NtHSR203J, *Nicotiana tabacum *HSR203J, AAF62404.1; PsHSR203j, *Pisum sativum *HSR203J, BAA85654.1; PsE86, *P. sativum *E86, BAA85654.1; GeHIDM, *Glycyrrhiza echinata *HIDM, BAD80839.1; GmHIDH, *Glycine max *HIDH, BAD80840.1; PrMC3, *Pinus radiata *MC3, AAD04946.2; VvBIG8.1, *Vitis vinifera *BIG8.1, >AF48726-1. Percentage bootstrap values (1000 bootstrap replicates) for groupings are given by each branch.

In *Actinidia *19 CXEs were identified, 18 of which are full length (Fig. 4). These include CXE members likely to encode gibberellin receptors (AdCXE9, AdCXE17, AdCXE15), an isoflavanone dehydratase (AeCXE11) and an ortholog of a plant defense-associated CXE (AcCXE12). Other CXEs from *Actinidia *align well with *Arabidopsis *CXEs and can be associated with the classes identified elsewhere [41]. The structure of an *Actinidia *CXE is now available (AeCXE1), the first plant CXE to have its structure solved [42]. AeCXE1 can hydrolyze a range of substrates from C2 to C16 esters, with a preference for C4 moieties. There are two CXEs from *Actinidia *(AdCXE16, AaCXE2) that may be orthologs to AeCXE1 from *A. eriantha*.

#### Terpene biosynthesis

**Figure 5:**
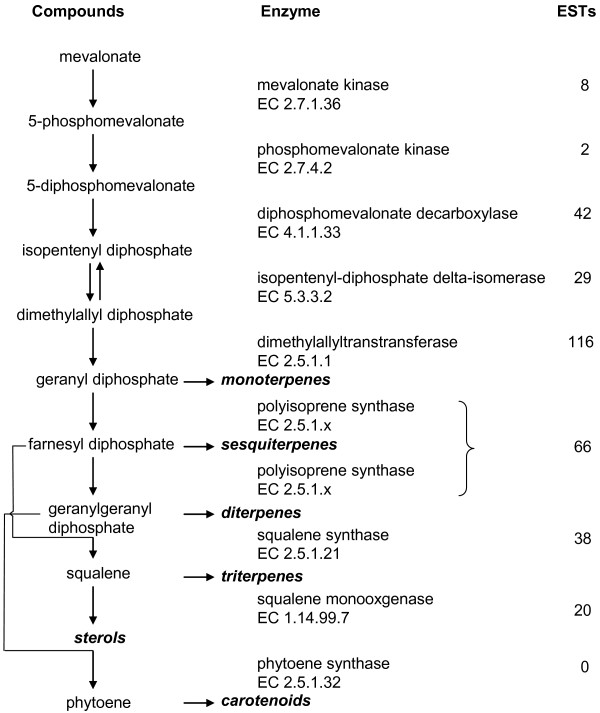
Distribution of *Actinidia *ESTs in the terpene biosynthesis pathway. Previously published sequences in GenBank belonging to the terpene biosynthetic pathway from mevalonate [43] were used to identify genes in the *Actinidia *EST database using BLAST searches. ESTs refers to the number of ESTs found in the *Actinidia *EST database for each step of the pathway.

*Actinidia *terpenoid compounds such as monoterpenes (C10), sesquiterpenes (C15) diterpenes (C20), triterpenes (C30), carotenoids, sterols, phytols and quinones are derived from two common precursor molecules, isopentenyl diphosphate and dimethylallyl diphosphate. These precursors are produced via the cytoplasmic mevalonate pathway or the chloroplastic 1-deoxy-D-xylulose-5-phosphate (mevalonate-independent) pathway [43]. Genes for enzymes in both these pathways are present in the *Actinidia *EST database, with the results for analysis of ESTs in the early steps of the mevalonate pathway shown in Fig. 5. The conversion of these precursors into other intermediates of the terpenoid biosynthetic pathway, including geranyl diphosphate, farnesyl diphosphate, and geranylgeranyl diphosphate, is carried out by polyisoprene synthase genes (EC 2.5.1.x). Over 200 ESTs with sequence homology to known polyisoprene synthase genes were found in the *Actinidia *EST database including 116 ESTs with similarity to dimethyallyltranstransferase (EC 2.5.1.1), 66 ESTs with homology to known monoterpene and sesquiterpene synthases and 38 ESTs homologous to squalene synthase (EC 2.2.1.21). No ESTs for phytoene synthase (EC 2.5.1.32) were identified.

The EST database collection has been used to identify a multifunctional terpene synthase gene from *A. deliciosa *flowers that produces the sesquiterpene germacrene D and a range of other sesquiterpene products at lower abundance [44]. This gene is represented by 10 sequences in the EST database.

### Genes related to color

#### Chlorophyll catabolism

Green fleshed commercial kiwifruit such as *A. deliciosa *'Hayward' are characterized by the retention of chlorophylls in the flesh of ripe fruit [45]. In contrast, the newer yellow-fleshed fruit cultivars (e.g. *A. chinensis *'Hort16A' and 'Jintao') degrade the chlorophyll during ripening and the consequent loss of the green color uncovers the underlying yellow carotenoid pigmentation.

**Figure 6:**
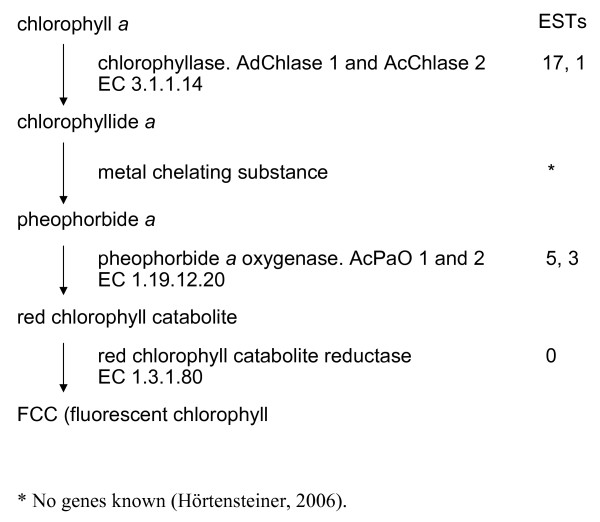
Distribution of *Actinidia *ESTs in the chlorophyll breakdown pathway. Previously published sequences in GenBank belonging to the chlorophyll breakdown pathway of higher plants [46] were used to identify genes in the *Actinidia *EST database using BLAST searches. ESTs refers to the number of ESTs found in the *Actinidia *EST database for each step of the pathway. Where more than one number of ESTs is given, each number represents ESTs that matched a different gene for that step.

The degradation of chlorophylls (leading to the production of colorless breakdown products) is controlled by catabolic enzymes in the chlorophyll degradation pathway [46]. *Actinidia *ESTs discovered in the pathway of chlorophyll breakdown are shown in Fig. 6. Chlorophyllase is the first enzyme of the catabolic pathway and it removes the phytol chain from chlorophyll *a*. Homologs of this enzyme have been mainly identified in libraries of vegetative tissues (breaking buds, leaves and petals) of *A. deliciosa *and *A. chinensis*. Homologs of pheophorbide *a *oxygenase have also been identified, with eight ESTs representing two genes from fruit libraries of *A. chinensis*. This enzyme is responsible for opening the tetrapyrrolic ring of the chlorophyll molecules, the critical step for the loss of green color. Surprisingly, we did not identify any ESTs of red chlorophyll catabolite reductase, an enzyme associated with pheophorbide *a *oxygenase and required to complete the porphyrin ring opening and the degradation of chlorophyll.

#### Carotenoid biosynthesis

**Figure 7:**
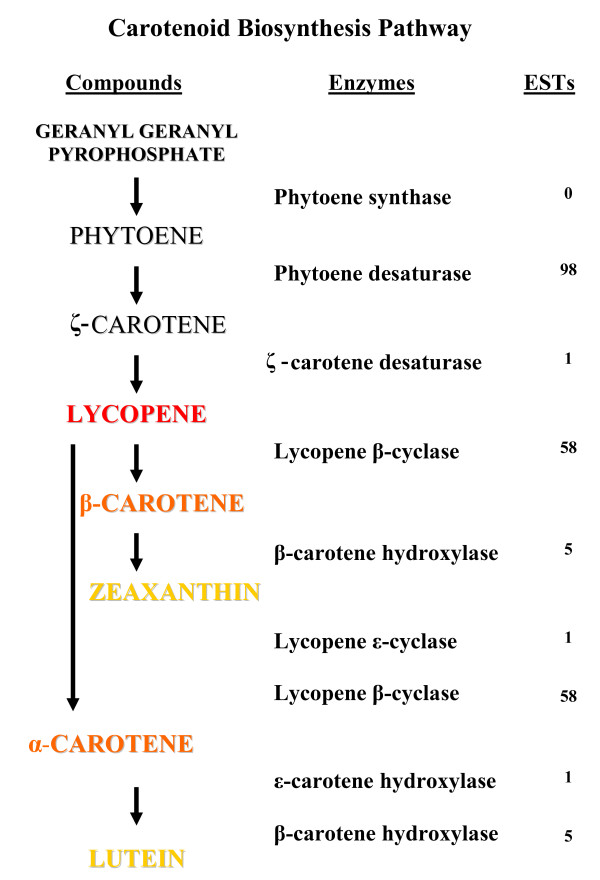
Distribution of *Actinidia *ESTs in the carotenoid biosynthetic pathway. Previously published sequences in GenBank belonging to the carotenoid biosynthetic pathway [47] were used to identify genes in the *Actinidia *EST database using BLAST searches. ESTs refers to the number of ESTs found in the *Actinidia *EST database for each step of the pathway.

The fruit of many *Actinidia *species contain a range of carotenoid pigments, including β-carotene and lutein [45]. The yellow-fleshed kiwifruit *A. chinensis *'Hort16A' develops as a green-fleshed fruit, but on ripening the chlorophyll is degraded exposing the carotenoids in the yellow flesh. ESTs for most steps in the biosynthesis of carotenoids [47-49] are present in the *Actinidia *EST database (Fig. 7), except the first step, phytoene synthase. Ninety eight ESTs for phytoene desaturase were identified, with 94 coming from *A. deliciosa *libraries, and the remainder from *A. chinensis *libraries. Of these 98 ESTs, 90 came from a single *A. deliciosa *fruit library with eight from bud and meristem libraries. Of the 56 ESTs found for lycopene β-cyclase, 23 were from petal tissues and 32 from fruit libraries. These ESTs were largely found in libraries from *A. deliciosa *(31) and *A. chinensis *(24), with only one EST from *A. eriantha*. In contrast, only a single EST was found for lycopene ε-cyclase, which is the enzyme required with lycopene β-cyclase to convert lycopene to α-carotene [50]. There were five ESTs of β-carotene hydroxylase found in petal (3), leaf (1) and dormant bud (1) libraries, but only a single EST was present for ε-carotene hydroxylase. Both of these enzymes are required for lutein synthesis [51].

#### Flavonoid and anthocyanin biosynthesis

Flavonoids are polyphenolic secondary metabolites synthesized through the phenylpropanoid pathway [52]. Anthocyanins are a subgroup of the flavonoids, which appear red to blue, depending on the pH, due to additions to their phenolic rings. In red fruit of *A. chinensis *and *A. deliciosa*, the predominant anthocyanins are cyanidin-based and the preferred glycosylation is in the 3' position [53]. While many of the kiwifruit libraries came from tissue with low levels of anthocyanins, several libraries were derived from tissue containing anthocyanins, including red fruits, brown skins and pink petals, leaves and buds (Additional file 2a).

**Figure 8:**
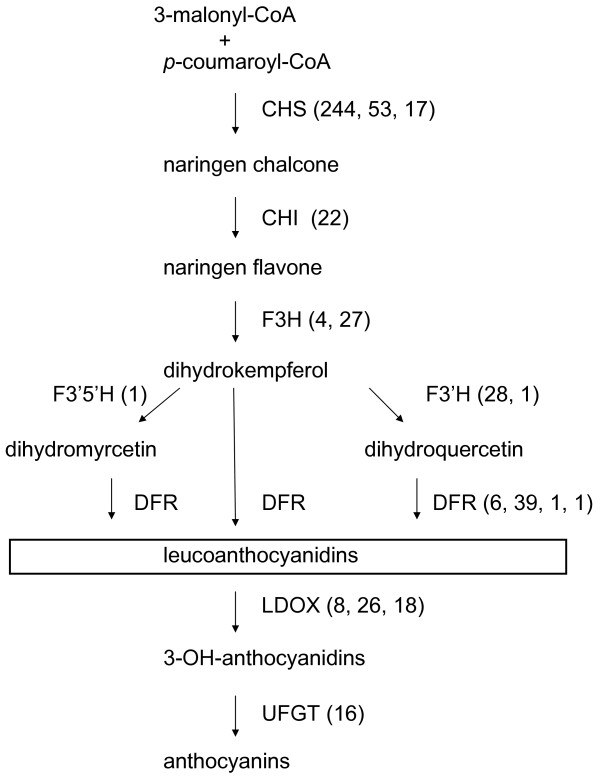
Distribution of *Actinidia *ESTs in the flavonoid biosynthetic pathway. Previously published sequences in GenBank belonging to the flavonoid biosynthetic pathway [49] were used to identify genes in the *Actinidia *EST database using BLAST searches. Enzyme names are followed in brackets by the number of ESTs in different families of genes encoding these enzymes. See text for abbreviations.

The anthocyanin biosynthetic pathway was analyzed by BLAST searching for ESTs representing different enzymes in the pathway (Fig. 8). Chalcone synthase (CHS) was a highly represented gene family in the EST database with ~300 ESTs. There appeared to be three distinct CHS NRs, possibly representing three genes, that are found in mostly young *A. eriantha *fruit libraries, as well as in bud and petal libraries. There was only one apparent chalcone isomerase (CHI) NR and over half the ESTs for this gene were found in shoot and bud libraries. Two distinct flavanol 3-hydroxylase (F3H) NRs were found, with ESTs predominantly found in bud and cell culture libraries. Only one flavonoid 3',5'-hydroxylase (F3'5'H) NR was found, from *A. eriantha *fruit, while ESTs for two distinct flavonoid 3'-hydroxylase (F3'H) NRs were predominantly found in shoot and bud libraries. Of the four potential dihydroflavonol reductase (DFR) NRs, the most highly represented was found in bud and young fruit. Three NRs, perhaps representing three gene families, of leucoanthocyanidin dioxygenase (LDOX) were found in the EST database, with most ESTs in bud libraries. The ESTs with highest sequence similarity to UDP-glucose flavonoid 3-O-glucosyltransferase (UFGT) are also predominantly found in developing buds of *A. deliciosa*.

Many of the ESTs for anthocyanin biosynthesis were sequenced from young fruit libraries (particularly *A. eriantha*) and in *A. deliciosa *buds. Developing buds from *A*. *deliciosa *appear to be a darker red color and therefore presumably accumulate more anthocyanins than buds from *A. chinensis *and *A. eriantha*, so it is not surprising to find ESTs for these later biosynthetic steps in bud tissue from *A. deliciosa*. However, ESTs for genes encoding enzymes from the early steps in the flavonoid pathway (e.g. CHS and F3H) are found in unpigmented *A. eriantha *fruit suggesting these tissues may accumulate a different class of flavonoids.

Most of the enzymes for anthocyanin biosynthesis are well represented in the EST database except for F3'5'H. This is not surprising given the only anthocyanins identified in *A. deliciosa *and *A. chinensis *are glycosylated in the 3' position. There are, however, species within the genus *Actinidia*, such as *A. melanandra*, that accumulate high levels of delphinidin, a different aglycone that would require F3'5'H activity. No ESTs from these species were sequenced.

### Genes related to healthful components

#### Ascorbic acid-related genes

**Figure 9:**
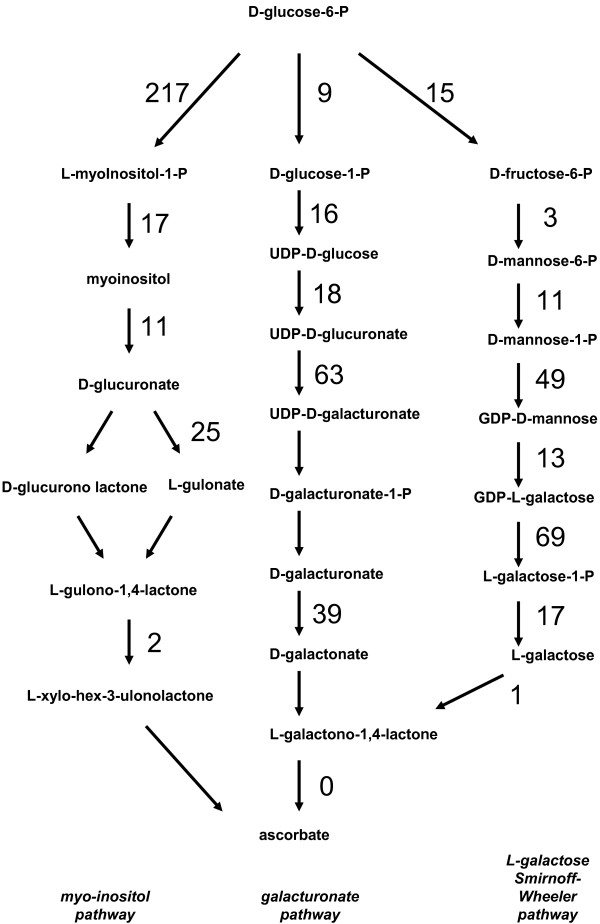
Distribution of *Actinidia *ESTs in three ascorbic acid biosynthetic pathways. Previously published sequences in GenBank belonging to the three known ascorbic acid biosynthetic pathways in plants [55] were used to identify genes in the *Actinidia *EST database using BLAST searches. The number of ESTs found in the Actinidia EST database for each step of each pathway is shown. Steps where no ESTs are noted have not had genes identified.

Kiwifruit are well known to be high in ascorbic acid (vitamin C), with values in commercial varieties ranging from 80 to 120 mg per 100 g fresh weight of fruit [54], and with *A. eriantha *having up to 10 times as much again. Ascorbic acid is synthesized in plants from glucose, through three potential pathways: the L-galactose pathway, the galacturonate pathway and through *myo*-inositol [55]. *Myo*-inositol is a major sugar alcohol found in kiwifruit (Additional file 7; [56,57]). The only pathway that is well established is the L-galactose pathway for which all the enzymes have now been identified in plants [55,58]. Many of the genes are known for the other two potential pathways, but several steps are still unknown. The *Actinidia *EST database was searched for ESTs similar to genes known to encode steps in these pathways. The numbers of ESTs homologous to various identified genes in the three pathways of ascorbic acid biosynthesis are shown in Fig. 9. Surprisingly for these high vitamin C plants, while the number of ESTs found in early steps in all the pathways is relatively large, the number of ESTs found in the two last steps is much lower, with no examples of ESTs encoding the last step of two of the pathways, that catalyzed by L-galactono lactone dehydrogenase.

#### Quinic acid metabolism

Fruit from *Actinidia *species have a relatively high total acid content (1–3% w/w) of which 40–60% can be quinic acid, 40–60% citric acid and 10% malic acid (Additional file 7). Quinic acid comprises an even higher proportion of the total acids during early fruit development [59], and over 25-fold variation in quinic acid content can be found in fruit of a single *A. chinensis *cross [60]. Kiwifruit are unusual in having such a high content of quinic acid as a fresh fruit, although levels of quinic acid in cranberry juice can be around 1% [61] and peaches can have up to 0.2% quinic acid [62].

**Figure 10:**
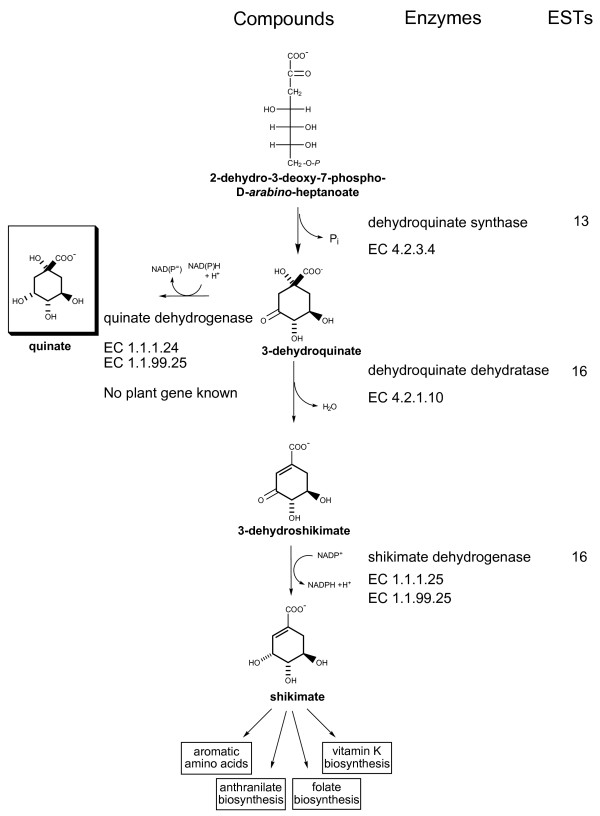
Distribution of *Actinidia *ESTs in the quinate biosynthetic pathway. Previously published sequences in GenBank belonging to the quinate biosynthetic pathway [98] were used to identify genes in the *Actinidia *EST database. The number of ESTs found in the *Actinidia *EST database for each step of each pathways is shown. Dehydroquinate dehydratase and shikimate dehydrogenase are two activities of a single bifunctional enzyme encoded by one gene [99].

Quinic acid is a key intermediate in lignin biosynthesis, folic acid metabolism, aromatic acid synthesis, anthranilate biosynthesis, and purine metabolism. The molecular and enzymatic control of quinic acid storage and metabolism may affect all these pathways. However, little is known about quinic acid metabolism in *Actinidia *species. ESTs for all the enzymes in the quinic acid biosynthetic pathway to shikimate (Fig. 10), except for a quinate dehydrogenase, have been identified in the *Actinidia *EST database.

Of the 13 ESTs identified for dehydroquinate synthase, eight were found in fruit or petal libraries, the rest were sequenced from bud or leaf libraries. Of the 16 ESTs identified for the bifunctional dehydroquinate dehydratase/shikimate dehydrogenase, 11 were from fruit or petal libraries and five from bud or leaf libraries. These ESTs were found mainly in libraries made from *A. deliciosa *and *A. chinensis *tissues and to a lesser extent in *A. arguta *and *A. eriantha *libraries, reflecting the distribution of ESTs among the species. An enzyme has been reported with predominantly quinate dehydrogenase activity [63], but the gene for this enzyme has not been cloned.

#### Allergens

Food allergies are estimated to affect ~6% of young children and ~3% of adults [64]. Kiwifruit have been recognized as a potentially allergenic fruit for over 20 years and reported allergies to kiwifruit are increasing [65]. Most allergy symptoms to kiwifruit are quite mild, but severe reactions have been reported, particularly in young children [66]. Allergies have been recorded in all three commercial species of *Actinidia *[67].

The *Actinidia *EST database contains sequences with homology to many known plant allergen proteins [68]. Potential allergens include the 2S albumin proteins, lipid transfer proteins, thaumatin-like protein (TLP), α-amylase/trypsin inhibitors, latex allergens, plant chitinases, profilins, cystatins, Bet v 1 homologous proteins and plant seed globulin allergens. However, only a small number of potential kiwifruit allergens have been directly confirmed by immunological testing, including the cysteine protease actinidin (Act d 1; [69]), an unidentified 43 kDa protein (Act d 2; [70], a TLP [70] and kiwellin [25].

ESTs for actinidin occurred throughout the 37 libraries from seven species that make up the database, and these clustered into acidic and basic forms of 10 distinct genes/alleles [22]. No matches were found by BLAST searching the EST database for the 43 kDa protein Act d 2. Kiwellin ESTs were found in libraries from most *Actinidia *species but were particularly abundant in *A. eriantha *ripe fruit skin, accounting for ~40% of ESTs in that library. The EST database contained 24 NRs with homology to TLPs from other plants (e.g. Pru a 2 from cherry and thaumatin 1 from *Thaumatococcus daniellii*), and all but one EST contained the 16 conserved cysteine residues characteristic of this protein class [71]. The various NRs could be aligned and clustered into 4 or 5 groups (data not shown), with two NRs accounting for the bulk of the ESTs (46 and 29%).

Globulin proteins (11S and 7S) have been reported from a wide range of seeds, including cereals [72,73] and legumes [74]. Globulin-like proteins are also found in *A. thaliana *[75] and a gene family of 10 globulin-like genes are present in *Arabidopsis *gene databases (The *Arabidopsis *Information Resource (TAIR), ). 11S globulins are composed of sets of polymorphic subunits derived from a multigene family. They exist as hexamers with molecular mass between 300 and 400 kDa, or as trimers. 11S globulin subunits consist of two polypeptide chains linked by at least one S-S bridge between cysteine residues at highly conserved positions in the acidic alpha-chain and basic beta-chain. Both chains are post-translationally generated from a common precursor protein, which represents the product of one member of the multigene family [76]. 11S globulins have been shown to be related to the 7S globulins through sequence comparisons [77].

**Figure 11:**
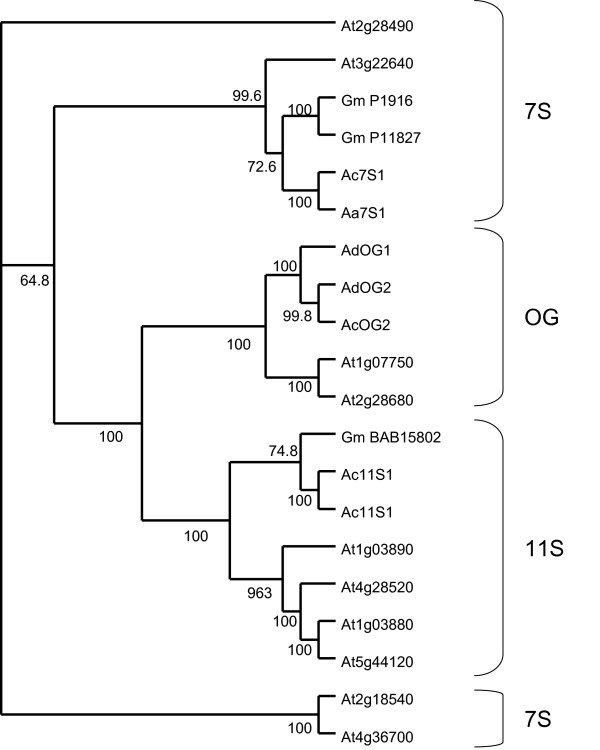
Phylogenetic tree of plant globulins of known function and *Actinidia *members of the globulin family. Previously published plant globulin sequences from GenBank were used to identify genes from the *Actinidia *EST database using BLAST searches. Abbreviations for species are as follows: Aa, *Actinidia arguta*; Ac, *A. chinensis*; Ad, *A. deliciosa*; At, *Arabidopsis thaliana*; Gm is *Glycine max *(soybean) followed by the Genbank accession number. Percentage bootstrap values (1000 bootstrap replicates) for groupings are given by each branch.

A BLAST search of the *Actinidia *EST sequence database with a range of previously identified globulin protein sequences identified two ESTs with a predicted protein sequence matching 11S globulin, both from a library made from *A. chinensis *whole fruit, which included seeds. Other related sequences from *Actinidia *fruit libraries belonged to the 7S vicilins-like family (38 ESTs), which were found in only fruit-based libraries from *A. deliciosa*, *A. chinensis *and *A. arguta*. These libraries would have all included seeds. A third grouping of sequences was identified that we have named other globulins (OG) (201 ESTs). The OG group appears to be more closely related to the 7S globulins than to the 11S (Fig 11). All three groupings are supported by the clustering of the *Arabidopsis *globulin genes as well as globulin genes from other species. While most OG ESTs were found in fruit libraries (81%), which included seeds, some members of the OG family were found in fruit skin, buds and petal libraries, suggesting a role other than as seed storage proteins. These OG ESTs were found in libraries derived from *A. deliciosa *(100), *A. chinensis *(42), *A. arguta *(39), and *A. eriantha *(16).

### Genes related to fruit softening

Some kiwifruit cultivars exhibit outstanding storage characteristics e.g. *A. deliciosa *'Hayward' fruit can be stored for 4–6 months at 0°C. Other *Actinidia *species exhibit a range of ripening and softening behaviors [78]. *A. eriantha *develops a peelable skin as the fruit ripen, *A. arguta *ripens in less than 10 days at 20°C versus 20–25 days for *A. deliciosa *and *A. chinensis *[79], whilst some small-fruited *Actinidia *genotypes tend to remain firm even towards the end of the ripening process [78].

**Figure 12:**
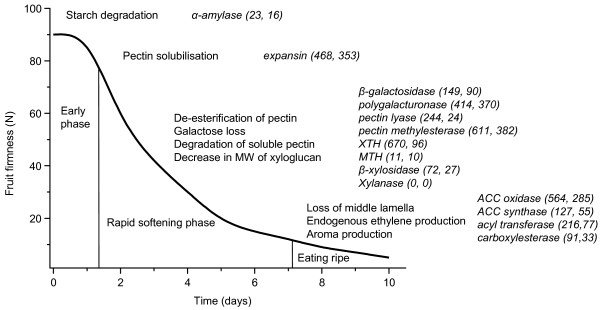
Distribution of *Actinidia *ESTs involved in postharvest kiwifruit softening. Schematic representation of postharvest kiwifruit softening in relation to the timing of key events in the softening process of ethylene-treated 'Hayward' fruit. Diagram modified from [80]). Previously published sequences in GenBank involved in postharvest softening processes were used to identify genes in the *Actinidia *EST database using BLAST searches. The first number in each set of brackets represents the total number of ESTs found in the *Actinidia *database and the second number is the number of ESTs found in fruit libraries.

The main chemical changes occurring in the cell wall during kiwifruit softening are pectin solubilization and degradation, reduction in the molecular weight of xyloglucan, and galactose loss from pectin side chains. Microscopically, the cell wall shows extensive swelling, until at the end of the softening process dissolution of middle lamellae can be observed [80]. These processes eventually lead to disintegration of the cell wall. Although similar chemical changes occur in other fruit [81], these changes occur concurrently with ethylene production and the respiratory climacteric. In kiwifruit, however, most of the softening process is well separated temporally from the climacteric and ethylene production (shown schematically in Fig. 12).

The genes for many enzymes involved in the key chemical changes in the cell wall during ripening are represented in the *Actinidia *EST database. Three enzymes involved in pectin degradation (pectin lyase, pectin methylesterase and polygalacturonase) are particularly abundant with 244, 611 and 414 ESTs, respectively. Genes encoding expansin, a protein implicated in pectin solubilization early in the kiwifruit softening process [80], are also highly abundant (468 ESTs). Galactose loss from fruit cell walls during the softening process has been attributed to the action of β-galactosidases [82]. A β-galactosidase from ripe kiwifruit has been purified. However, the activity of the enzyme *in vitro *against synthetic or purified native substrates from kiwifruit was far too low to account for the amount of galactose loss observed during softening [83]. Ninety β-galactosidase ESTs were observed in fruit libraries in the EST database and are good candidates for genetic manipulation and to study the role of this enzyme. During softening, xyloglucan is also hydrolyzed. As xyloglucan and cellulose create the major load-bearing network of the cell wall, any xyloglucan degradation is thought to weaken the cell wall, resulting in softening of the fruit. In kiwifruit, hydrolysis of xyloglucan occurs mainly during the rapid softening phase. Endoglucanases and xyloglucan transglucosylase/hydrolase (XTH) enzymes are implicated in this process. XTHs catalyze both hydrolytic and transglucosylation reactions, and an XTH enzyme capable of carrying out both reactions has been isolated from kiwifruit [84]. This particular XTH, however, is expressed late in kiwifruit ripening, when xyloglucan degradation has already come to an end. The 96 XTH ESTs found in fruit libraries will allow the identification of genes involved in hydrolysis of xyloglucan during the rapid softening phase. ESTs for enzymes that act on minor kiwifruit cell wall components such as galactoglucomannans or glucuronoarabinoxylans [80] were less abundant in the EST database (e.g. mannan transglycosylase/hydrolase (MTH), 10 ESTs found in fruit).

## Discussion

We report on a significant resource of over 130,000 ESTs derived from a range of *Actinidia *species (Table 1). We targeted tissues and developmental stages in order to sample genes involved in physiological and biochemical processes including fruit ripening, flavor development, control of color and the synthesis of chemicals with health-related attributes. For this reason, the two most widely cultivated species of kiwifruit, *A. chinensis *and *A. deliciosa*, are well represented with together over 100,000 ESTs (Table 1). In addition, fruit and bud libraries are also well represented, with over 38,000 and 50,000 ESTs, respectively. *A. chinensis *and *A. deliciosa *are so closely related, as is *A. setosa *(Li) C.F. Liang et A.R.Ferguson, that they are variously treated as being distinct species or as varieties of the one species. The other two main species studied, *A. arguta *and *A. eriantha *Benth., also have commercial potential but are more distantly related [2].

The genus *Actinidia *is unusual in how much inter-taxal and intra-taxal variation in ploidy and in the wild, there is a structured reticulate pattern of diploids, tetraploids, hexaploids, and octoploids in diminishing frequency, associated, in at least some taxa, with geographic separation of ploidy races. *A. deliciosa *is hexaploid, *A. setosa *is diploid, and there are diploid and tetraploid races of *A. chinensis*, the tetraploids apparently coming from a restricted part of the natural distribution of the species. Most evidence suggests that diploid *A. chinensis *was a progenitor of tetraploid *A. chinensis *and hexaploid *A. deliciosa *but it is not clear whether genomes from other species have contributed. The basic chromosome number (n = 29) is high and it seems increasingly likely that diploid *A. chinensis *is itself a rediploidized palaeopolyploid [2].

As is common in EST sequencing projects (e.g., [6,8,10], there is a high degree of redundancy in the ESTs, with clustering reducing the number of unique sequences from over 132,000 to 41,858 NRs (18,070 TCs, 23,788 singletons). We would expect this number of NRs to be an overestimate of the number of genes in *Actinidia*, especially given that the database contains sequences from multiple species of *Actinidia*. Using the same correction used in the apple EST paper [10], we expect an *Actinidia *genome to have around 27,000 genes.

On average 20% (± 2% standard error) of the sequences from each library with over 1000 ESTs were singletons suggesting a high degree of novelty in these libraries. On average 28% (± 4%) of sequences did not have a homolog in the various public databases based on BLAST searches with an E value > 1.0e^-10^. An average of 16% (± 3%) of ESTs were identified as 3' UTR candidates based on the presence of a poly(A) tail within 40 bp of the start (taking into account reverse sequences). These 3' sequences would not be expected to be identified by BLAST searches and so would affect the novelty of a library. Less than 12% of NRs did not have BLAST matches (E > 10) in the *Arabidopsis *proteome, Uniref, NCBI ref or SwissProt databases.

There was only a small degree of overlap in NRs between libraries. Libraries from different species and different tissues showed a 5 to 9% overlap in NRs, libraries from different species but the same tissue showed an 8 to 10% overlap and libraries from the same species but different tissue showed a 7 to 13% commonality in NRs. These comparisons were made over five large libraries with more than 9,000 EST members each and an average of 2.1 ESTs per NR. These results suggest that there were more NRs in common between libraries made from the same tissue or from the same species, but this tendency was not particularly marked.

Detecting SNPs using an automatically assembled EST database is a cost effective way to discover new DNA polymorphisms and develop novel markers, although it can be a challenging task, especially in polyploid *Actinidia *species. A significant proportion of the sequence variants predicted from overlapping ESTs within an NR will correspond to "real" SNPs, which means the sequence differences found are allelic variants of a given locus and not sequencing errors or differences between paralogs, homoeologs or orthologs. Homoeolog SNPs could be particularly common in the polyploid accessions of species such as *A. deliciosa *and *A. arguta *that make up a large proportion of this database, but are also possible in diploids as a result of conserved gene pairs of paleopolyploid origin. Allelic SNPs can be used directly and converted into molecular markers for genetic mapping, population genetics and linkage disequilibrium studies or for marker-assisted selection. A SNP marker for determining the sex of kiwifruit seedlings [85] has already been successfully utilized. Since the database contains sequence data from multiple species, and ~40% of TCs are made up of more than one species, several SNPs were detected in the *Actinidia *EST database corresponding to sequence between orthologous loci from different *Actinidia *species. Hence, they cannot fully be considered as allelic SNPs, but more as species-specific variations. However, as kiwifruit breeding programs often use controlled crosses between different species, the interspecific SNPs will segregate in the progeny and be useful as markers.

The incidence of SSRs in NRs was higher in *Actinidia *(30%) than in apple (20%), and the frequency of di-nucleotide and tri-nucleotide SSRs differed between these two species. This increase was evident in all of the sequence classes but greatest in AG and AC (double the incidence among apple NRs). Even though the *Actinidia *genome EST resource represents several species, while apple came mainly from one species, this would not explain these differences. Perhaps the longer period of domesticity in apple, based on a narrow genetic basis compared to kiwifruit, may explain the difference. Alternatively it may reflect that a greater proportion of homoeologs have grouped into TCs in the polyploid kiwifruit data than in the apple dataset.

Overall the codon usage of the three *Actinidia *species shares many similarities with that of other dicotyledons represented in the codon usage database [86]. Comparisons with *Arabidopsis *codon usage showed that *A. deliciosa *and *A. eriantha *differ markedly for 15 and 17 amino acids, respectively, whereas *A. chinensis *differs in its preference for eight particular amino acids. Further comparisons with apple, grape, pear, peach, loblolly pine, tomato, citrus, potato and tobacco showed that the codon preference of the *Actinidia *species is most similar to that of apple [10]. *A. deliciosa *differs from apple only in its codon preference for aspartate, glycine, isoleucine and leucine. *A. eriantha *also differs from apple for these four amino acids and also serine. The codon preference for *A. chinensis *and apple also differ for only four amino acids, these being asparagine, glutamine, threonine and valine. CpG suppression is also evident in *Actinidia *species with an XCG/XCC ratio of between 0.68 and 0.71 for the three species evaluated. This modest level of suppression of the CpG di-nucleotides is similar to that of apple (0.64) and differs markedly from that of *Arabidopsis *which shows nearly no suppression (0.92) and from the high level found in grape (0.35). This may well reflect different levels of methylation in the coding sequences used by different species of plants.

Mapman was used to assign function to the *Actinidia *NRs and thus to their constitutive ESTs. Only 32% of the ESTs did not have an *Arabidopsis *homolog at E < 1.0e^-10^. In general, the functional distribution of NRs was very similar to the functional distribution of *Arabidopsis *proteins (Table 3) suggesting that the sampling of *Actinidia *ESTs well represented the major functional classes of plant genes. This is surprising given the biased selection of libraries with virtually no root ESTs sequenced. However, the high number of bud meristem libraries meant that genes expressed in metabolically active dividing tissue were sampled.

Fruit of the *Actinidia *genus show several characteristics that distinguish them from other fruit species. These include flesh color (green is the most common, but yellow, orange and red fruit also occur in the genus), chemical composition including high vitamin C and quinic acid contents, and a novel aroma composition (Additional file 7), characterized by the abundant esters. In addition, kiwifruit has been identified as a fruit with a potential to cause allergenicity among consumers, although this is a problem common to many other fruit. For this reason, we analyzed the *Actinidia *EST database to identify genes involved in these pathways and products. These analyses showed the depth and usefulness of the database for selecting candidate genes for most steps in the selected pathways. The other useful characteristic of the *Actinidia *EST database is the wide range of genetic and phenotypic diversity sampled across the *Actinidia *genus (Fig. 1) and the value of using this diversity to discover novel traits through functional genomics and through mapping and positional cloning approaches.

## Conclusion

This paper describes an EST resource in the *Actinidia *genus and discusses many of the properties of this collection. However, there is still a tremendous challenge in understanding the molecular basis of the genetic diversity of this genus, and we expect putting this EST resource into the public domain will enhance future understanding of the genetic basis of the many divergent traits in this fruit.

## Methods

### Plant material

Tissues were collected from *Actinidia *species growing in New Zealand at HortResearch research orchards in Auckland, Bay of Plenty (Te Puke) and Northland (Kerikeri) from 1999 to 2003. Table 1 and Additional file 2a provide details of tissues, species and treatments.

### Library construction and EST sequencing

Total RNA was extracted from *Actinidia *tissues by established methods [87,88]. Messenger RNA was isolated from total RNA by passage through oligo(dT)-cellulose columns (GE Healthcare, USA), and cDNA cloned into either phage (ZapcDNA Synthesis Kit and Zap-cDNA Gigapack III Gold Cloning Kit; Stratagene, USA) or plasmid-based libraries (SuperScript System for cDNA Synthesis and Cloning; Invitrogen, USA). In some libraries, subtractive or other enrichment techniques were used (Additional file 2b).

Plasmids from the phage cDNA libraries were mass excised, according to the manufacturer's recommendations (Stratagene). Plasmid extractions were then undertaken on individual bacterial colonies of either the phage-derived or the plasmid-derived cDNA libraries and the corresponding cDNA inserts sequenced predominantly from the 5' end. Big Dye Terminator sequencing reactions were resolved on ABI377, ABI3100, or ABI3700 sequencers, according to the manufacturer's instructions (Applied Biosystems, USA). For determination of the complete sequence of cDNA clones, M13R and M13F or T3 and T7 primers were used for 5' and 3' end sequencing. EST-specific primers were used to determine the complete sequence of cDNA clones. In situations where EST clones had long poly(A) tails (generally 40 nucleotides) and, therefore, failed to yield good quality sequence with standard sequencing primers, an anchored T24VN primer was used. Resulting sequences were edited manually and assembled using Sequencher software, version 4.0.5 (GeneCodes). Sequencing progress for each cDNA library was assessed manually for clone length, redundancy and sequence quality.

### Bioinformatics

EST sequences were automatically trimmed of vector, adapter, and low quality sequence regions, and uploaded to a relational database. Automatic annotation was performed using the HortResearch BioView sequence annotation pipeline (BioView – an enterprise bioinformatics system for automated analysis and annotation of non-genomic DNA sequence (Crowhurst R, Davy M, Deng C, unpublished)) that utilizes a relational database (MySQL; ). The EST clustering phase was performed using The Institute for Genomic Research (TIGR) gene indices clustering tools . The representation of protein families, domains, and functional sites within the *Actinidia *NRs was determined using InterProScan [89]. The proteome for *Arabidopsis *(*Arabidopsis thaliana*) was obtained from TAIR [90], and comparisons to proteins from *Arabidopsis *using BLASTx were used to identify *Actinidia *NRs with similarity to *Arabidopsis *proteins.

Detection of SSRs was undertaken using a PERL program within BioView that identified tandem repetition of sequence words in target sequences. SSRs were characterized by repeat type (di-, tri-, or tetra-nucleotide repeat units), repeat length, and position. Only repeats longer than 10 bp were included in the analyses. When reporting the frequency of repeat classes, different di- and tri-nucleotide sequences were combined by type; for example, AG repeats also encompassed repeats identified as GA and their complementary sequences CT or TC repeats. Prediction of SNPs and insertion/deletions and sequencing errors was performed using PERL scripts within BioView that parsed the output of contig sequences generated by the CAP3 DNA sequence assembly program [91].

Codon usage tables were derived from cDNA sequences encoding predicted full-length proteins. Clones were predicted to be full length only if they started with an ATG codon and terminated with a stop codon at positions equivalent to those of other plant genes. Codon usage was calculated from sequences using the CUSP program implemented within EMBOSS [92]. For functional analysis using Mapman [27], each *Actinidia *NR was assigned the nearest *Arabidopsis *BLASTx match (E < 1.0e^-10^) if there was no conflicting evidence from domains or families detected by InterProScan. The NR and its EST members were then assigned a Mapman classification bin and bin name based on that assigned to the *Arabidopsis *match.

### Homology searching and phylogenetic analysis

Previously published protein sequences from GenBank were used to identify genes in the *Actinidia *EST database using tBLASTn searches [93], typically with a cutoff value of E < 1.0e^-20^. Identified genes were then manually checked to ensure accuracy. Amino acid alignments of predicted proteins were constructed using Clustal X. Phylogenetic analysis was carried out using the PHYLIP suite of programs [94]. Distances were calculated using protdist, and the Fitch method was used to construct a tree. Bootstrap analysis was conducted using 1000 bootstrap replicates using seqboot [94]. Treeview (v.1.6.6) was used to display resulting trees [95].

### Microarray construction

For each predicted NR in the *Actinidia *EST database, a 45–55 mer oligonucleotide was created, using PERL scripts within BioView that selected all possible oligonucleotides within a gene based on uniqueness of sequence, lack of repetitive regions, and a constant melting temperature. For each NR the most 3' oligonucleotide that passed these criteria was selected for synthesis. As the EST libraries were sequenced from different *Actinidia *species, the resulting oligonucleotides were derived from genes from different *Actinidia *species. A total of 17,472 oligonucleotides were made, with the majority coming from *A. chinensis *and *A. deliciosa*. A summary of the oligonucleotides used to construct the *Actinidia *microarray slides by library and species is given in Additional file 4. Genomic DNA from *A. deliciosa *'Hayward' and *A. eriantha *(genotype 11-6-15e) was labeled and each microarray hybridized as described previously [12]. Data were normalized using quantile normalization in the Bioconductor package Limma [96].

### Chemical analysis of actinidia flowers and fruit

#### Volatile flavor and aroma compounds

Both headspace and solvent sampling was used to obtain volatiles. For headspace sampling of flowers, whole flowers (2.5–5 g) that were 50–75% open were placed in a 250 ml Quickfit™ Erlenmeyer flask to which was fitted a volatile trapping cartridge packed with 100 mg of Chromosorb 105™ absorbent and sampled and analyzed as described earlier [29]. For fruit, ten fruit (eating-ripe) were cut longitudinally in half and one half of each fruit was used for headspace sampling and analysis [29]. The other halves were frozen in liquid N_2 _for solvent extraction.

For solvent extraction, either ~1 g of flower petals were rinsed in 2 ml of purified 50:50 pentane/Et_2_O or 20 g of fruit was pulped, and gently shaken in a sealed test tube with 10 mL of pentane:Et_2_O (8:2) for 1–2 min, four times over a 2 h period at room temperature. Samples were processed and analyzed by GC-MS as described earlier [29].

Quantification of compounds was carried out using an average FID response based on methyl butanoate, ethyl butanoate, hexanol and methyl benzoate. Component identification was by comparison with spectra in the Mass Spectral Database (1998 NIST and an in-house database), retention indices (in-house database) and in some cases direct GC-MS comparison with authentic standards.

#### Analysis of fruit acids and sugars

Tissue was ground in liquid N_2 _and acids and sugars were extracted from a known mass of tissue (1.4–2.5 g) into 10 mL of 80% EtOH (with adonitol and tartaric acid added as internal standards) at 60°C for 1 h. The extracts were processed, sugars and organic acids derivatized and then quantified by GC [97]. Identification of the acids and sugars was confirmed by GC-MS comparison with mixtures of authentic compounds.

#### Sequence data

Sequence data for the ESTs described in this paper can be found at GenBank under accession numbers FG396013 – FG528589.

## Authors' contributions

EFW, EHAR, EJFS, GSR, JHB, KCS, MDT, MW, RMD, and RS all contributed to bioinformatics analysis. ACA, CAD, KBM, LLB, MM, MR, RDN, RGA, RPH, and SMB all undertook bioinformatics analysis and manuscript drafting. RNC undertook bioinformatics programming, HLB, and KK, did chemical analysis. AJM did chemical analysis and interpretation and manuscript writing. APG and EAM undertook design and manuscript drafting. BJJ, BN, DW, EG, KRL, MYW, RE, RW, YKY, and YYW did the EST sequencing and clone selection. ACR, ARF, LGF and MAN provided basic genetic material and analysis and manuscript drafting. WAL did manuscript drafting and bioinformatics analysis. BU contributed significantly by doing Mapman analysis and manuscript drafting. MWD and RJS did microarray analysis and interpretation. DC did SNP analysis and manuscript writing.

## Supplementary Material

Additional file 1 Additional Table 1. Expressed sequence tags (ESTs) sequenced in Angiosperm orders and families. Click here for file

Additional file 2 Additional Table 2a. Summary of library names, descriptions and statistics for sequenced *Actinidia *ESTs. Additional Table 2b. *Actinidia *libraries that were subtracted before sequencing to reduce redundant sequences. Click here for file

Additional file 3 Additional Table 3 Codon usage calculated using 707 full-length *Actinidia *cDNA sequences. Click here for file

Additional file 4 Additional Table 4. Source of oligonucleotides used to construct the *Actinidia *microarray slides by library and species Click here for file

Additional file 5 Additional Table 5. Highly expressed ESTs in the *Actinidia *EST database. Click here for file

Additional file 6 Additional Table 6. Most frequent InterPro families found in *Actinidia *NRs. Click here for file

Additional file 7 Additional Table 7. Volatiles, acids, and sugars in *Actinidia *flowers and fruit Click here for file
