# Supplementary material for: Analysis of expressed sequence tags from Actinidia: applications of a cross species EST database for gene discovery in the areas of flavor, health, color and ripening
Source: BMC Genomics. 2008 Jul 27;9:351. doi: 10.1186/1471-2164-9-351 (PMC2515324; doi:10.1186/1471-2164-9-351)
Supplement: Additional file 1 — Additional Table 1. Expressed sequence tags (ESTs) sequenced in Angiosperm orders and families. [file 1471-2164-9-351-S1.doc]

Additional Table 1. Expressed sequence tags (ESTs) sequenced in Angiosperm orders and families. Data was taken from GenBank in October 2007

| References | Classification | | Common Name | # ESTs |
| --- | --- | --- | --- | --- |
| **Eudicotyledons** |  | |  |  |
|  | **Brassicales** | |  | **2,171,356** |
|  | Brassicaceae | | Mustard family | 2,171,289 |
|  | *Arabidopsis thaliana* | |  | 1,279,945 |
|  | *Brassica rapa* | |  | 180,611 |
|  | *Brassica napus* | |  | 567,240 |
|  | *Brassica oleracea* | |  | 59,736 |
|  |  | |  |  |
|  | **Fabales** | |  | **983,221** |
|  | Fabaceae | | Pea family | 983,221 |
| Unpublished | *Glycine max* | | Soybean | 392,337 |
| Unpublished | *Arachis hypogaea* | | Peanut | 54,913 |
| [1] | *Arachis stenosperma* | | Wild peanut | 6,264 |
| [2] | *Phaseolus vulgaris* | | Bean | 22,847 |
| [3] | *Trifolium repens* | | White clover | 46 |
|  |  | |  |  |
|  | **Solanales** | |  | **862,318** |
|  | Convolvulaceae | | Morning glory family | 83,157 |
|  | *Ipomoea batatas* | | Sweet potato | 19,468 |
|  | *Ipomoea nil* | | Japanese morning glory | 62,282 |
|  | Solanaceae | | Nightshade family | 779,161 |
|  | *Nicotiana benthamiana* | |  | 41,440 |
|  | *Nicotiana tabacum* | | Common tobacco | 157,600 |
| [4] | *Petunia x hybrida* | | Garden petunia | 14,017 |
|  | *Capsicum annuum* | |  | 31,090 |
| [5] | *Solanum lycopersicum* | | Tomato | 258,408 |
| [6] | *Solanum tuberosum* | | Potato | 227,375 |
|  |  | |  |  |
|  | **Asterales** | |  | **805,678** |
|  | Asteraceae | | Daisy family | 804,808 |
|  | *Helianthus annuus* | | Common sunflower | 94,111 |
| [7] | *Centaurea maculosa* | |  | 44,925 |
| [8] | *Stevia rebaudiana* | |  | 5,548 |
|  |  | |  |  |
|  | **Malpighiales** | |  | **555,226** |
|  | Euphorbiaceae | | Spurge family | 157,522 |
| [9] | *Ricinus communis* | | Castor bean | 53,402 |
|  | *Euphorbia esula* | | Leafy spurge | 47,543 |
| [10] | *Manihot esculenta* | | Cassava | 36,120 |
|  | Salicaceae | | Willow family | 369,657 |
| [11, 12] | *Populus* | | Poplars | 369,237 |
|  |  | |  |  |
|  | **Rosales** | |  | **391,529** |
|  | Rosaceae | | Rose family | 380,936 |
| Unpublished | *Prunus persica* | | Peach | 70,972 |
| [13] | *Malus x domestica* | | Apple | 255,103 |
|  |  | |  |  |
|  | **Vitales** | |  | **363,377** |
|  | Vitaceae | | Grape family | 363,377 |
| [14-16] | *Vitis vinifera* | | Wine grape | 336,365 |
|  |  | |  |  |
|  | **Malvales** | |  | **289,106** |
|  | Malvaceae | | Mallow family | 289,080 |
|  | *Gossypium hirsutum* | | Upland cotton | 177,182 |
|  | *Gossypium raimondii* | |  | 63,577 |
|  |  | |  |  |
|  | **Sapindales** | |  | **225,497** |
|  | Rutaceae | | Rue family | 225,364 |
|  | *Citrus sinensis* | |  | 94,738 |
|  | *Citrus clementina* | |  | 62,250 |
|  |  | |  |  |
|  | **Ranunculales** | |  | **94,358** |
|  | Ranunculaceae | | Buttercup family | 85,076 |
|  | *Aquilegia formosa x A. pubescens* |  | | 85,039 |
| [17] | *Eschscholzia californica* | | California poppy | 9,083 |
|  |  | |  |  |
|  | **Lamiales** | |  | **90,982** |
|  | Lamiaceae | | Mint family | 35,835 |
| [18] | *Stenogyne rugosa* | |  | 666 |
|  | *Ocimum basilicum* | | Sweet basil | 23,260 |
|  | Plantaginaceae | | Speedwell family | 25,313 |
|  | *Antirrhinum majus* | | Snapdragon | 25,310 |
|  |  | |  |  |
|  | **Caryophyllales** | |  | **88,295** |
|  | Aizoaceae | | Iceplant family | 27,385 |
|  | *Mesembryanthemum crystallinum* | | Common iceplant | 27,348 |
|  | Amaranthaceae | | Amaranth family | 29,479 |
|  | *Beta vulgaris* | | Beet | 26,745 |
|  |  | |  |  |
|  | **Gentianales** | |  | **76,279** |
|  | Rubiaceae | | Madder family | 67,233 |
|  | *Coffea canephora* | | Robusta coffee | 55,692 |
|  |  | |  |  |
|  | **Fagales** | |  | **26,082** |
|  | Juglandaceae | | Walnut family | 17,994 |
|  | *Juglans hindsii x Juglans regia* |  | | **12794** |
|  |  | |  |  |
|  | **Myrtales** | |  | **15,740** |
|  | Myrtaceae | |  | 15,731 |
|  | *Eucalyptus* | |  | 15,240 |
|  |  | |  |  |
|  | **Ericales** | |  | **11,413** |
|  | Ericaceae | |  | 6,375 |
|  | Theaceae | |  | **2293** |
| [19] | *Camellia sinensis* | | Tea | 2,277 |
|  |  | |  |  |
| **Liliopsida (monocotyledons)** |  | |  |  |
|  | **Poales** | |  | **4,667,627** |
|  | Poaceae | | Grass family | 4,661,977 |
| [20] | *Zea mays* | | Corn | 1,271,325 |
| Various | *Oryza sativa* | | Rice | 1,211,418 |
| [21] | *Triticum aestivum* | | Bread wheat | 1,049,881 |
| [22] | *Hordeum vulgare* | | Barley | 461,874 |
| [23] | *Saccharum* | | Sugar cane | 255,964 |
| [24] | *Sorghum bicolor* | | Sorghum | 204,308 |
| Unpublished | *Sorghum propinquum* | |  | 20,881 |
| [25] | *Secale cereale* | | Rye | 9,293 |
| [26] | *Pennisetum glaucum* | |  | 2,848 |
| [27] | *Lolium perenne* | | Ryegrass | 1,492 |
|  |  | |  |  |
|  | **Asparagales** | |  | **59,310** |
|  | Alliaceae | |  | 20,201 |
| [28] | *Allium cepa* | | Onion | 20,159 |

1. Proite K, Leal-Bertioli SC, Bertioli DJ, Moretzsohn MC, da Silva FR, Martins NF, Guimaraes PM: **ESTs from a wild *Arachis* species for gene discovery and marker development**. *BMC Plant Biol* 2007, **7**(1):7.

2. Ramirez M, Graham MA, Blanco-Lopez L, Silvente S, Medrano-Soto A, Blair MW, Hernandez G, Vance CP, Lara M: **Sequencing and analysis of common bean ESTs. Building a foundation for functional genomics**. *Plant Physiol* 2005, **137**(4):1211-1227.

3. Sawbridge T, Ong E-K, Binnion C, Emmerling M, Meath K, Nunan K, O'Neill M, O'Toole F, Simmonds J, Wearne K: **Generation and analysis of expressed sequence tags in white clover (*Trifolium repens* L.)**. *Plant Science* 2003, **165**(5):1077-1087.

4. Shimamura K, Ishimizu T, Nishimura K, Matsubara K, Kodama H, Watanabe H, Hase S, Ando T: **Analysis of expressed sequence tags from Petunia flowers**. *Plant Science* 2007, **173**(5):495-500.

5. Fei Z, Tang X, Alba RM, White JA, Ronning CM, Martin GB, Tanksley SD, Giovannoni JJ: **Comprehensive EST analysis of tomato and comparative genomics of fruit ripening**. *Plant J* 2004, **40**(1):47-59.

6. Ronning CM, Stegalkina SS, Ascenzi RA, Bougri O, Hart AL, Utterbach TR, Vanaken SE, Riedmuller SB, White JA, Cho J *et al*: **Comparative analyses of potato expressed sequence tag libraries**. *Plant Physiol* 2003, **131**(2):419-429.

7. Broz AK, Broeckling CD, He J, Dai X, Zhao PX, Vivanco JM: **A first step in understanding an invasive weed through its genes: an EST analysis of invasive *Centaurea maculosa***. *BMC Plant Biol* 2007, **7**(1):25.

8. Brandle JE, Richman A, Swanson AK, Chapman BP: **Leaf Ests from *Stevia rebaudiana*: a resource for gene discovery in diterpene synthesis**. *Plant Mol Biol* 2002, **50**(4-5):613-622.

9. Lu C, Wallis JG, Browse J: **An analysis of expressed sequence tags of developing castor endosperm using a full-length cDNA library**. *BMC Plant Biology* 2007, **7**:42.

10. Lopez C, Jorge V, Piégu Bit, Mba C, Cortes D, Restrepo S, Soto M, Laudié M, Berger C, Cooke R *et al*: **A unigene catalogue of 5700 expressed genes in cassava**. *Plant Molecular Biology* 2004, **56**(4):541-554.

11. Kohler A, Delaruelle C, Martin D, Encelot N, Martin F: **The poplar root transcriptome: analysis of 7000 expressed sequence tags**. *FEBS Lett* 2003, **542**(1-3):37-41.

12. Sterky F, Bhalerao RR, Unneberg P, Segerman B, Nilsson P, Brunner AM, Charbonnel-Campaa L, Lindvall JJ, Tandre K, Strauss SH *et al*: **A *Populus* EST resource for plant functional genomics**. *PNAS* 2004, **101**(38):13951-13956.

13. Newcomb RD, Crowhurst RN, Gleave AP, Rikkerink EHA, Allan AC, Beuning LL, Bowen JH, Gera E, Jamieson KR, Janssen BJ *et al*: **Analyses of expressed sequence tags from apple**. *Plant Physiol* 2006, **141**(1):147-166.

14. Keilin T, Pang X, Venkateswari J, Halaly T, Crane O, Keren A, Ogrodovitch A, Ophir R, Volpin H, Galbraith D *et al*: **Digital expression profiling of a grape-bud EST collection leads to new insight into molecular events during grape-bud dormancy release**. *Plant Science* 2007, **173**(4):446-457.

15. Moser C, Segala C, Fontana P, Salakhudtinov I, Gatto P, Pindo M, Zyprian E, Toepfer R, Grando MS, Velasco R: **Comparative analysis of expressed sequence tags from different organs of *Vitis vinifera* L**. *Funct Integr Genomics* 2005, **5**(4):208-217.

16. Peng FY, Reid KE, Liao N, Schlosser J, Lijavetzky D, Holt R, Martinez Zapater JM, Jones S, Marra M, Bohlmann J *et al*: **Generation of ESTs in *Vitis vinifera* wine grape (Cabernet Sauvignon) and table grape (Muscat Hamburg) and discovery of new candidate genes with potential roles in berry development**. *Gene* 2007, **402**(1-2):40-50.

17. Carlson J, Leebens-Mack J, Wall P, Zahn L, Mueller L, Landherr L, Hu Y, Ilut D, Arrington J, Choirean S *et al*: **EST database for early flower development in California poppy (*Eschscholzia californica* Cham., Papaveraceae) tags over 6000 genes from a basal eudicot**. *Plant Molecular Biology* 2006, **62**(3):351-369.

18. Lindqvist C, Scheen AC, Yoo MJ, Grey P, Oppenheimer DG, Leebens-Mack JH, Soltis DE, Soltis PS, Albert VA: **An expressed sequence tag (EST) library from developing fruits of an Hawaiian endemic mint (*Stenogyne rugosa*, Lamiaceae): characterization and microsatellite markers**. *BMC Plant Biol* 2006, **6**(1):16.

19. Park J-S, Kim J-B, Hahn B-S, Kim K-H, Ha S-H, Kim J-B, Kim Y-H: **EST analysis of genes involved in secondary metabolism in *Camellia sinensis* (tea), using suppression subtractive hybridization**. *Plant Science* 2004, **166**(4):953-961.

20. Fernandes J, Brendel V, Gai X, Lal S, Chandler VL, Elumalai RP, Galbraith DW, Pierson EA, Walbot V: **Comparison of RNA expression profiles based on maize expressed sequence tag frequency analysis and micro-array hybridization**. *Plant Physiol* 2002, **128**(3):896-910.

21. Ramalingam J, Pathan MS, Feril O, Ross K, Ma XF, Mahmoud AA, Layton J, Rodriguez-Milla MA, Chikmawati T, Valliyodan B *et al*: **Structural and functional analyses of the wheat genomes based on expressed sequence tags (ESTs) related to abiotic stresses**. *Genome* 2006, **49**(10):1324-1340.

22. Michalek W, Weschke W, Pleissner KP, Graner A: **EST analysis in barley defines a unigene set comprising 4,000 genes**. *Theor Appl Genet* 2002, **104**(1):97-103.

23. Vettore AL, da Silva FR, Kemper EL, Souza GM, da Silva AM, Ferro MI, Henrique-Silva F, Giglioti EA, Lemos MV, Coutinho LL *et al*: **Analysis and functional annotation of an expressed sequence tag collection for tropical crop sugarcane**. *Genome Res* 2003, **13**(12):2725-2735.

24. Jang CS, Kamps TL, Skinner DN, Schulze SR, Vencill WK, Paterson AH: **Functional classification, genomic organization,putatively cis-acting regulatory elements, and relationship to quantitative trait loci, of sorghum genes with rhizome-enriched expression**. *Plant Physiol* 2006, **142**(3):1148-1159.

25. Milla MA, Butler E, Huete AR, Wilson CF, Anderson O, Gustafson JP: **Expressed sequence tag-based gene expression analysis under aluminum stress in rye**. *Plant Physiol* 2002, **130**(4):1706-1716.

26. Mishra R, Reddy P, Nair S, Markandeya G, Reddy A, Sopory S, Reddy M: **Isolation and characterization of expressed sequence tags (ESTs) from subtracted cDNA libraries of *Pennisetum glaucum* seedlings**. *Plant Molecular Biology* 2007, **64**(6):713-732.

27. Sawbridge T, Ong E-K, Binnion C, Emmerling M, McInnes R, Meath K, Nguyen N, Nunan K, O'Neill M, O'Toole F: **Generation and analysis of expressed sequence tags in perennial ryegrass (*Lolium perenne* L.)**. *Plant Science* 2003, **165**(5):1089-1100.

28. Kuhl JC, Cheung F, Yuan Q, Martin W, Zewdie Y, McCallum J, Catanach A, Rutherford P, Sink KC, Jenderek M *et al*: **A unique set of 11,008 onion expressed sequence tags reveals expressed sequence and genomic differences between the monocot orders Asparagales and Poales**. *Plant Cell* 2004, **16**(1):114-125.
