# Supplementary material for: Analysis of expressed sequence tags from Actinidia: applications of a cross species EST database for gene discovery in the areas of flavor, health, color and ripening
Source: BMC Genomics. 2008 Jul 27;9:351. doi: 10.1186/1471-2164-9-351 (PMC2515324; doi:10.1186/1471-2164-9-351)
Supplement: Additional file 2 — Additional Table 2a. Summary of library names, descriptions and statistics for sequenced Actinidia ESTs. Additional Table 2b. Actinidia libraries that were subtracted before sequencing to reduce redundant sequences. [file 1471-2164-9-351-S2.doc]

Additional Table 2a. Summary of library names, descriptions and statistics for sequenced *Actinidia* ESTs.

| Library Code | Library description | Minimum length | Average length | Maximum length | No. EST sequences | No. Singletons | % of singletons |
| --- | --- | --- | --- | --- | --- | --- | --- |
| KAAA | *Actinidia deliciosa** developing shoot buds | 50 | 450 | 785 | 9472 | 1896 | 20.0 |
| KABA | *A. deliciosa* petal (all stages) | 50 | 458 | 794 | 9950 | 1973 | 19.8 |
| KACA | *A. deliciosa* ripe fruit outer cortex, three months stored, firmness range 2.8-9.1N | 59 | 356 | 659 | 74 | 15 | 20.3 |
| KADA | *A. deliciosa* ripe fruit inner cortex, three months stored, firmness range 2.8-9.1N | 50 | 374 | 744 | 9798 | 2564 | 26.2 |
| KAEA | *A. deliciosa* dormant buds before hydrogen cyanamide treatment | 210 | 461 | 658 | 82 | 18 | 22.0 |
| KAEB | *A. deliciosa* dormant buds before hydrogen cyanamide treatment | 195 | 536 | 743 | 1013 | 342 | 33.8 |
| KAFB | *A. deliciosa* buds 3 days after hydrogen cyanamide treatment | 105 | 543 | 771 | 4585 | 1103 | 24.1 |
| KAGA | *A. hemsleyana* ‘Kaimai’ thick storage roots (non-woody parts) | 199 | 461 | 688 | 326 | 43 | 13.2 |
| KAHA | *A.* *arguta* ‘K2D4’ petal, all stages | 115 | 566 | 709 | 1117 | 124 | 11.1 |
| KAHB | *A.* *arguta* ‘K2D4’ petal, all stages | 230 | 603 | 744 | 83 | 36 | 43.4 |
| KAHC | *A.* *arguta* ‘K2D4’ petal, all stages | 194 | 520 | 756 | 636 | 240 | 37.7 |
| KAIA | *A.* *chinensis** mixed ripe fruit | 103 | 570 | 768 | 7004 | 1422 | 20.3 |
| KAJA | *A.* *eriantha* 11-4-18a ripe fruit skin | 103 | 558 | 730 | 1118 | 127 | 11.4 |
| KAJB | *A.* *eriantha* 11-4-18a ripe fruit skin | 199 | 527 | 747 | 1090 | 163 | 15.0 |
| KAKA | *A. deliciosa* dormant buds one day before hydrogen cyanamide treatment | 65 | 524 | 754 | 4689 | 538 | 11.5 |
| KALA | *A. deliciosa* dormant buds three days after hydrogen cyanamide treatment | 50 | 520 | 784 | 9196 | 1657 | 18.0 |
| KALB | Transcription factor enriched library from KALA library | 206 | 564 | 735 | 82 | 2 | 2.4 |
| KALC | Transcription factor enriched library from KALA library | 252 | 548 | 721 | 81 | 0 | 0.0 |
| KALD | Transcription factor enriched library from KALA library | 202 | 594 | 740 | 78 | 1 | 1.3 |
| KAMA | *A.* *hemsleyana* ‘Kaimai’ minor roots with nematodes | 105 | 531 | 746 | 4775 | 153 | 3.2 |
| KANA | *A.* *setosa* non woody stem | 122 | 492 | 695 | 1020 | 207 | 20.3 |
| KAOA | *A.* *arguta ‘*K2D4’ vine ripened fruit | 194 | 545 | 774 | 901 | 210 | 23.3 |
| KAOB | *A.* *arguta* ‘K2D4*’* vine ripened fruit | 225 | 531 | 683 | 89 | 25 | 28.1 |
| KAPA | *A.* *eriantha* EA01_01 (43-03-14b) male petal | 95 | 537 | 786 | 778 | 185 | 23.8 |
| KAPB | *A.* *eriantha* EA01_01 (43-03-14b) male petal | 212 | 601 | 748 | 81 | 25 | 30.9 |
| KAPC | *A.* *eriantha* EA01_01 (43-03-14b) male petal | 194 | 566 | 796 | 529 | 164 | 31.0 |
| KAQA | *A.* *polygama* PC01_04 (48-03-09c) male petal | 149 | 545 | 768 | 661 | 250 | 37.8 |
| KAQB | *A.* *polygama* PC01_04 (48-03-09c) male petal | 227 | 576 | 773 | 85 | 39 | 45.9 |
| KAQC | *A.* *polygama* PC01_04 (48-03-09c) male petal | 193 | 577 | 750 | 602 | 234 | 38.9 |
| KASA | *A.* *chinensis* MP060 and MP104 mapping population range of fruit ripeness (red centre) | 202 | 547 | 728 | 82 | 13 | 15.9 |
| KASB | *A.* *chinensis* KASA library subtracted with MP060 | 203 | 569 | 746 | 915 | 123 | 13.4 |
| KATA | *A.* *indochinensis* IA01_01 48-04-17b petal | 313 | 545 | 714 | 74 | 27 | 36.5 |
| KAUA | *A.* *chinensis* CK51-05 petal | 217 | 579 | 738 | 215 | 71 | 33.0 |
| KAUB | *A.* *chinensis* CK51-05 petal | 177 | 533 | 757 | 806 | 183 | 22.7 |
| KAUC | *A.* *chinensis* CK51-05 petal | 213 | 584 | 762 | 40 | 11 | 27.5 |
| KAVA | *A.* *chinensis* cell culture, exponential growth | 233 | 565 | 727 | 40 | 8 | 20.0 |
| KAVB | *A.* *chinensis* cell culture, exponential growth | 212 | 629 | 769 | 120 | 31 | 25.8 |
| KAVC | *A.* *chinensis* cell culture, exponential growth | 201 | 562 | 756 | 4691 | 138 | 2.9 |
| KAWA | *A.* *chinensis* breaking buds | 351 | 586 | 691 | 44 | 10 | 22.7 |
| KAWB | *A.* *chinensis* breaking buds | 150 | 597 | 795 | 733 | 166 | 22.6 |
| KAWC | *A.* *chinensis* breaking buds | 59 | 562 | 798 | 14912 | 2169 | 14.5 |
| KAXA | *A.* *chinensis* MP056 versus MP060 mapping populations | 202 | 557 | 729 | 84 | 16 | 19.0 |
| KAYA | *A.* *chinensis* young leaf library | 199 | 531 | 700 | 84 | 19 | 22.6 |
| KAYB | *A.* *chinensis* young leaf library | 95 | 525 | 797 | 4152 | 1060 | 25.5 |
| KAYC | *A.* *chinensis* young leaf library | 100 | 530 | 761 | 2403 | 386 | 16.1 |
| KAZA | *A.* *chinensis* young fruit library (two sizes) | 217 | 512 | 683 | 163 | 26 | 16.0 |
| KAZB | *A.* *chinensis* young fruit library (two sizes) | 12 | 539 | 789 | 9775 | 1902 | 19.5 |
| KAZC | *A.* *chinensis* young fruit library (two sizes) | 84 | 676 | 833 | 371 | 42 | 11.3 |
| KAZD | *A.* *chinensis* young fruit library (two sizes) | 128 | 742 | 955 | 377 | 87 | 23.1 |
| KAZE | *A.* *chinensis* young fruit library (two sizes) | 114 | 742 | 943 | 368 | 79 | 21.5 |
| KHFA | *A. arguta* ‘K2D4’ vine ripened fruit | 53 | 531 | 762 | 3764 | 351 | 9.3 |
| KHFB | *A. arguta* ‘K2D4’ vine ripened fruit | 202 | 601 | 763 | 667 | 182 | 27.3 |
| KSFA | *A. deliciosa* small fruit 13 days after anthesis | 50 | 456 | 747 | 3410 | 511 | 15.0 |
| KUBA | *A. deliciosa* breaking bud | 50 | 320 | 745 | 5241 | 1071 | 20.4 |
| KUFA | *A. eriantha* 11-4-18a young fruit | 50 | 463 | 771 | 9051 | 1350 | 14.9 |
|  | Weighted averages | 77 | 503 | 772 |  |  | 17.9 |
|  | Totals |  |  |  | 132577 | 23788 | 17.9 |

* All *A. deliciosa* were ‘Hayward’ and all *A. chinensis* were ‘Hort16A’ unless otherwise noted.

Additional Table 2b. *Actinidia* libraries that were subtracted before sequencing to reduce redundant sequences.

| **Library before subtraction** | **Library after subtraction** | **Subtracted with** |
| --- | --- | --- |
| KAOB | KAOA | Acidic actinidin (Genbank accession No. X16466) |
| KHFA | KHFB | Acidic actinidin |
| KAJA | KAJB | ESTs 193469 and 193832 |
| Not sequenced | KAIA | *Actinidia deliciosa ‘K32’* cDNA and acidic actinidin |
| KASA | KASB | cDNA from MP060 |
| KAHB | KAHC | *A. deliciosa* cDNA used to make KABA |
| KAUA | KAUB, KAUC | *A. deliciosa* cDNA used to make KABA |
| KAPB | KAPA | *A. deliciosa* cDNA used to make KABA |
| KAQB | KAQA | *A. deliciosa* cDNA used to make KABA |
