# Supplementary material for: Analysis of expressed sequence tags from Actinidia: applications of a cross species EST database for gene discovery in the areas of flavor, health, color and ripening
Source: BMC Genomics. 2008 Jul 27;9:351. doi: 10.1186/1471-2164-9-351 (PMC2515324; doi:10.1186/1471-2164-9-351)
Supplement: Additional file 3 — Additional Table 3 Codon usage calculated using 707 full-length Actinidia cDNA sequences. [file 1471-2164-9-351-S3.doc]

Additional Table 3. Codon usage calculated using 707 full-length *Actinidia* cDNA sequences. Codon usage calculated using the CUSP program from EMBOSS [1].

|  |  | *A. deliciosa* |  |  | *A. chinensis* | |  |  | *A. eriantha* |  |  |
| --- | --- | --- | --- | --- | --- | --- | --- | --- | --- | --- | --- |
| Codon | Amino acid | Fraction^a^ | /1000^b^ | Number | Fraction^a^ | /1000^b^ | | Number | Fraction^a^ | /1000^b^ | Number |
| GCA | A | 0.21 | 15.6 | 1497 | 0.25 | 17.07 | | 2039 | 0.2 | 14.37 | 390 |
| GCC | A | 0.33 | 24.06 | 2309 | 0.27 | 18.05 | | 2156 | 0.33 | 24.14 | 655 |
| GCG | A | 0.17 | 12.27 | 1177 | 0.14 | 9.53 | | 1138 | 0.19 | 13.75 | 373 |
| GCT | A | 0.29 | 21.14 | 2029 | 0.34 | 22.94 | | 2740 | 0.28 | 20.12 | 546 |
| TGC | C | 0.58 | 9.76 | 937 | 0.56 | 10.05 | | 1200 | 0.66 | 11.83 | 321 |
| TGT | C | 0.42 | 7.22 | 693 | 0.44 | 8.04 | | 960 | 0.34 | 6.15 | 167 |
| GAC | D | 0.46 | 24.32 | 2334 | 0.41 | 21.78 | | 2602 | 0.51 | 27.34 | 742 |
| GAT | D | 0.54 | 28.57 | 2742 | 0.59 | 31.94 | | 3816 | 0.49 | 26.13 | 709 |
| GAA | E | 0.43 | 27.47 | 2636 | 0.45 | 28.35 | | 3387 | 0.4 | 23.47 | 637 |
| GAG | E | 0.57 | 36.19 | 3473 | 0.55 | 35.35 | | 4223 | 0.6 | 35.19 | 955 |
| TTC | F | 0.59 | 24.01 | 2304 | 0.51 | 20.53 | | 2453 | 0.59 | 26.86 | 729 |
| TTT | F | 0.41 | 16.71 | 1604 | 0.49 | 19.67 | | 2350 | 0.41 | 18.61 | 505 |
| GGA | G | 0.26 | 18.46 | 1772 | 0.27 | 19.34 | | 2310 | 0.26 | 20.23 | 549 |
| GGC | G | 0.26 | 18.87 | 1811 | 0.23 | 16.04 | | 1916 | 0.27 | 21.19 | 575 |
| GGG | G | 0.24 | 17.1 | 1641 | 0.23 | 16.3 | | 1956 | 0.25 | 19.27 | 523 |
| GGT | G | 0.24 | 17.64 | 1693 | 0.27 | 19.18 | | 2291 | 0.22 | 16.88 | 458 |
| CAC | H | 0.58 | 14.67 | 1408 | 0.5 | 12.75 | | 1523 | 0.66 | 17.47 | 474 |
| CAT | H | 0.42 | 10.83 | 1039 | 0.5 | 12.54 | | 1498 | 0.34 | 8.84 | 240 |
| ATA | I | 0.19 | 8.86 | 850 | 0.21 | 9.77 | | 1167 | 0.19 | 8.81 | 239 |
| ATC | I | 0.41 | 18.92 | 1816 | 0.36 | 16.52 | | 1974 | 0.44 | 20.8 | 565 |
| ATT | I | 0.4 | 18.52 | 1777 | 0.43 | 19.57 | | 2338 | 0.37 | 17.5 | 475 |
| AAA | K | 0.38 | 21.72 | 2084 | 0.39 | 22.53 | | 2691 | 0.36 | 20.64 | 560 |
| AAG | K | 0.62 | 35.11 | 3369 | 0.61 | 35.9 | | 4288 | 0.64 | 36.26 | 984 |
| CTA | L | 0.08 | 7.22 | 693 | 0.1 | 8.61 | | 1028 | 0.08 | 7.33 | 199 |
| CTC | L | 0.26 | 21.98 | 2109 | 0.21 | 18.12 | | 2165 | 0.28 | 24.98 | 678 |
| CTG | L | 0.16 | 13.45 | 1291 | 0.16 | 13.74 | | 1641 | 0.18 | 15.88 | 431 |
| CTT | L | 0.19 | 15.96 | 1532 | 0.21 | 18.3 | | 2186 | 0.18 | 16.36 | 444 |
| TTA | L | 0.08 | 6.83 | 655 | 0.08 | 7.52 | | 898 | 0.08 | 7.11 | 193 |
| TTG | L | 0.23 | 20.1 | 1929 | 0.24 | 21.22 | | 2535 | 0.2 | 18.57 | 504 |
| ATG | M | 1 | 24.56 | 2357 | 1 | 25.26 | | 3018 | 1 | 24.17 | 656 |
| AAC | N | 0.53 | 23.46 | 2251 | 0.48 | 21.99 | | 2627 | 0.57 | 24.17 | 656 |
| AAT | N | 0.47 | 21.25 | 2039 | 0.52 | 23.95 | | 2861 | 0.43 | 17.98 | 488 |
| CCA | P | 0.28 | 16.99 | 1630 | 0.31 | 18.53 | | 2213 | 0.27 | 14.3 | 388 |
| CCC | P | 0.22 | 13.11 | 1258 | 0.19 | 11.14 | | 1331 | 0.24 | 12.68 | 344 |
| CCG | P | 0.23 | 13.88 | 1332 | 0.2 | 11.77 | | 1406 | 0.26 | 13.86 | 376 |
| CCT | P | 0.27 | 16.05 | 1540 | 0.3 | 18.2 | | 2174 | 0.23 | 12.12 | 329 |
| CAA | Q | 0.5 | 18.44 | 1770 | 0.5 | 21.04 | | 2513 | 0.48 | 16.4 | 445 |
| CAG | Q | 0.5 | 18.66 | 1791 | 0.5 | 20.7 | | 2473 | 0.52 | 17.61 | 478 |
| AGA | R | 0.22 | 11.58 | 1111 | 0.25 | 13.23 | | 1581 | 0.22 | 10.87 | 295 |
| AGG | R | 0.27 | 14.3 | 1372 | 0.29 | 15.15 | | 1810 | 0.26 | 13.08 | 355 |
| CGA | R | 0.12 | 6.25 | 600 | 0.11 | 5.86 | | 700 | 0.11 | 5.6 | 152 |
| CGC | R | 0.15 | 7.76 | 745 | 0.11 | 5.79 | | 692 | 0.15 | 7.67 | 208 |
| CGG | R | 0.15 | 8.21 | 788 | 0.13 | 7.02 | | 839 | 0.17 | 8.62 | 234 |
| CGT | R | 0.09 | 5 | 480 | 0.11 | 5.69 | | 680 | 0.09 | 4.75 | 129 |
| AGC | S | 0.17 | 14.92 | 1432 | 0.15 | 13.75 | | 1642 | 0.18 | 15.15 | 411 |
| AGT | S | 0.12 | 10.96 | 1052 | 0.14 | 12.14 | | 1450 | 0.12 | 9.8 | 266 |
| TCA | S | 0.16 | 14.51 | 1392 | 0.18 | 16.27 | | 1943 | 0.14 | 11.76 | 319 |
| TCC | S | 0.19 | 17.28 | 1658 | 0.16 | 14.08 | | 1682 | 0.2 | 16.84 | 457 |
| TCG | S | 0.16 | 14.08 | 1351 | 0.14 | 12.11 | | 1447 | 0.16 | 13.52 | 367 |
| TCT | S | 0.2 | 17.62 | 1691 | 0.23 | 19.91 | | 2379 | 0.2 | 16.21 | 440 |
| ACA | T | 0.21 | 10.93 | 1049 | 0.25 | 12.45 | | 1487 | 0.22 | 11.24 | 305 |
| ACC | T | 0.35 | 18.01 | 1728 | 0.3 | 14.64 | | 1749 | 0.36 | 18.39 | 499 |
| ACG | T | 0.17 | 8.65 | 830 | 0.15 | 7.54 | | 901 | 0.18 | 9.54 | 259 |
| ACT | T | 0.27 | 13.57 | 1302 | 0.3 | 14.72 | | 1759 | 0.24 | 12.12 | 329 |
| GTA | V | 0.11 | 6.87 | 659 | 0.12 | 7.34 | | 877 | 0.11 | 7.41 | 201 |
| GTC | V | 0.24 | 14.87 | 1427 | 0.21 | 13.24 | | 1582 | 0.25 | 17.06 | 463 |
| GTG | V | 0.34 | 21.65 | 2078 | 0.32 | 20.02 | | 2392 | 0.35 | 23.99 | 651 |
| GTT | V | 0.31 | 19.28 | 1850 | 0.35 | 21.92 | | 2618 | 0.29 | 19.46 | 528 |
| TGG | W | 1 | 13.88 | 1332 | 1 | 14.08 | | 1682 | 1 | 15.22 | 413 |
| TAC | Y | 0.58 | 15.56 | 1493 | 0.53 | 14.08 | | 1682 | 0.62 | 18.17 | 493 |
| TAT | Y | 0.42 | 11.17 | 1072 | 0.47 | 12.41 | | 1483 | 0.38 | 11.06 | 300 |
| TAA | * | 0.36 | 1.14 | 109 | 0.32 | 0.86 | | 103 | 0.3 | 0.92 | 25 |
| TAG | * | 0.2 | 0.64 | 61 | 0.22 | 0.58 | | 69 | 0.25 | 0.77 | 21 |
| TGA | * | 0.44 | 1.38 | 132 | 0.46 | 1.23 | | 147 | 0.45 | 1.4 | 38 |

^a^ Proportion of usage of a given codon among its redundant set (i.e. the set of codons that code for that codon’s amino acid)

^b^ Codon frequency normalized per 1000 bases.

1. Rice P, Longden I, Bleasby A**: EMBOSS: The European Molecular Biology Open Software Sui**te*. Trends Gene*t 2000**,** 16:276–277.
