# Supplementary material for: Analysis of expressed sequence tags from Actinidia: applications of a cross species EST database for gene discovery in the areas of flavor, health, color and ripening
Source: BMC Genomics. 2008 Jul 27;9:351. doi: 10.1186/1471-2164-9-351 (PMC2515324; doi:10.1186/1471-2164-9-351)
Supplement: Additional file 4 — Additional Table 4. Source of oligonucleotides used to construct the Actinidia microarray slides by library and species [file 1471-2164-9-351-S4.doc]

Additional Table 4. Source of oligonucleotides used to construct the *Actinidia* microarray slides by library (A) and species (B)

(A)

| **Library** | **Species** | **No. Oligos** |
| --- | --- | --- |
| bud | *A. chinensis* | 2846 |
| bud | *A. deliciosa* | 4906 |
|  | **total bud** | **7752** |
| cell culture | *A. chinensis* | 289 |
|  | **total cells** | **289** |
| fruit | *A. arguta* | 320 |
| fruit | *A. chinensis* | 3902 |
| fruit | *A. deliciosa* | 1058 |
| fruit | *A. eriantha* | 762 |
|  | **total fruit** | **6042** |
| leaf | *A. chinensis* | 1641 |
|  | **total leaf** | **1641** |
| petal | *A. arguta* | 159 |
| petal | *A. chinensis* | 279 |
| petal | *A. deliciosa* | 642 |
| petal | *A. eriantha* | 216 |
|  | **total petal** | **1296** |
|  | other | **452** |
|  | **Total array** | **17,472** |

(B)

| **Species** | **Oligos** |
| --- | --- |
| *A. arguta* | 479 |
| *A. chinensis* | 8957 |
| *A. deliciosa* | 6606 |
| *A. eriantha* | 978 |
| *A. hemsleyana* | 148 |
| *A. indochinensis* | 12 |
| *A. polygama* | 188 |
| *A. setosa* | 104 |
