# Supplementary material for: Analysis of expressed sequence tags from Actinidia: applications of a cross species EST database for gene discovery in the areas of flavor, health, color and ripening
Source: BMC Genomics. 2008 Jul 27;9:351. doi: 10.1186/1471-2164-9-351 (PMC2515324; doi:10.1186/1471-2164-9-351)
Supplement: Additional file 5 — Additional Table 5. Highly expressed ESTs in the Actinidia EST database. [file 1471-2164-9-351-S5.doc]

Additional Table 5. Highly expressed ESTs in the *Actinidia* EST database.

Tentative Contig (TC) sequences with 40 or more EST members were selected for analysis using Mapman [1]. As some TCs represent the same gene, but map to different parts of the gene, groups of TCs with identical descriptions were compared and if they showed greatest identity to the same *Arabidopsis* gene, the number of ESTs was combined in these TCs. Subsequently, TCs with similar descriptions, but mapping to different *Arabidopsis* genes, also had their number of EST members combined (No. of related genes, including the TC in the row). Mapman codes and names were assigned on the basis of homology to *Arabidopsis* sequences as described in the Methods.

| TC description | No. of ESTs | No. of related ESTs | Mapman features | | | |
| --- | --- | --- | --- | --- | --- | --- |
|  | | | Bin code | Bin name | Secondary bin code | Secondary bin name |
| Cysteine proteinase | 219 | 1266 | 29.5.3 | protein degradation cysteine protease |  |  |
| Metallothionein | 126 | 769 | 15.2 | metal handling binding, chelation and storage |  |  |
| Ripening protein; Barwin endoglucanase related cluster | 527 | 730 | 35.2 | not assigned unknown |  |  |
| Elongation factor 1-alpha (EF-1-alpha) | 162 | 681 | 29.2.4 | protein synthesis elongation |  |  |
| Ubiquitin | 187 | 518 | 29.5.11 1 | protein degradation ubiquitin |  |  |
| Chlorophyll a-b binding protein | 239 | 475 | 1.1.1.1 | PS light reaction photosystem II LHC-II |  |  |
| Aquaporin membrane Plasma membrane intrinsic protein | 122 | 390 | 34.19.1 | transport major intrinsic proteins PIP |  |  |
| Peptidyl-prolyl cis-trans isomerase Cyclophilin | 136 | 334 | 31.3 | cell cycle |  |  |
| Heat shock protein 90 related cluster | 46 | 324 | 20.2.1 | stress abiotic heat |  |  |
| Polygalacturonase related cluster | 58 | 311 | 35.2 | not assigned unknown |  |  |
| Endochitinase | 93 | 304 | 20.1 | stress biotic |  |  |
| S-adenosylmethionine synthetase 2 | 174 | 285 | 13.1.3.4 | amino acid metabolism synthesis aspartate family methionine | 15 2 | metal handling binding, chelation and storage |
| Sucrose synthase 1 | 111 | 205 | 2.2.1.5 | major CHO metabolism degradation sucrose SUSY |  |  |
| Fructose-bisphosphate aldolase cytoplasmic isozyme | 50 | 192 | 1.3.6 | PS Calvin cycles aldolase | 4 7 | glycolysis aldolase |
| Nonspecific lipid transfer protein 2 precursor (LTP 2) | 48 | 186 | 11.6 | lipid metabolism lipid transfer proteins etc |  |  |
| Tubulin alpha-1 chain related cluster | 125 | 177 | 31.1 | cell organisation |  |  |
| S-adenosylmethionine synthetase 1 | 117 | 159 | 15.2 | metal handling binding, chelation and storage | 13 1 3 4 | amino acid metabolism synthesis aspartate family methionine |
| Subtilisin protease related cluster | 49 | 158 | 29.5.1 | protein degradation subtilases |  |  |
| Tubulin beta-4 chain related | 112 | 156 | 31.1 | cell organisation |  |  |
| Glutamine synthetase cytosolic isozyme | 83 | 138 | 15.2 | metal handling binding, chelation and storage |  |  |
| Phosphate-responsive 1 family protein | 43 | 128 | 35.2 | not assigned unknown |  |  |
| ATP synthase beta subunit 2 | 72 | 115 | 9.9 | mitochondrial electron transport / ATP synthesis F1-ATPase |  |  |
| Pectate lyase | 41 | 97 | 10.6.3 | cell wall degradation pectate lyases and polygalacturonases |  |  |
| Cysteine proteinase | 758 |  | 29.5.3 | protein degradation cysteine protease |  |  |
| Metallothionein | 547 |  | 15.2 | metal handling binding, chelation and storage |  |  |
| Xyloglucan endotransglucosylase/hydrolase protein | 405 |  | 10.7 | cell wall modification |  |  |
| Elongation factor 1-alpha (EF-1-alpha) | 393 |  | 29.2.4 | protein synthesis elongation |  |  |
| Hypothetical or unknown protein | 339 |  | 35.2 | not assigned unknown |  |  |
| Proline related protein | 311 |  | 35.2 | not assigned unknown |  |  |
| Hypothetical or unknown protein | 302 |  | 35.2 | not assigned unknown |  |  |
| Ubiquitin | 280 |  | 29.5.11.1 | protein degradation ubiquitin ubiquitin |  |  |
| Polygalacturonase (pectinase) | 253 |  | 10.6.3 | cell wall degradation pectate lyases and polygalacturonases |  |  |
| Chalcone synthase 1 | 241 |  | 16.8.2 | secondary metabolism flavonoids chalcones |  |  |
| Proline protein; protease inhibitor/seed storage/lipid transfer protein (LTP) family protein | 230 |  | 26.21 | misc protease inhibitor/seed storage/lipid transfer protein (LTP) family protein |  |  |
| 3-hydroxy-3-methylglutaryl-coenzyme A reductase 2 (HMG-CoA reductase 2) | 218 |  | 16.1.2.3 | secondary metabolism isoprenoids mevalonate pathway HMG-CoA reductase |  |  |
| Hypothetical or unknown protein | 218 |  | 15.2 | metal handling binding, chelation and storage |  |  |
| Heat shock protein 90 related cluster | 214 |  | 20.2.1 | stress abiotic heat |  |  |
| DNAJ heat shock protein | 212 |  | 20.2.1 | stress abiotic heat |  |  |
| Polyphenol oxidase B, chloroplast precursor | 212 |  | 35.2 | not assigned unknown |  |  |
| RuBisCO small subunit | 186 |  | 1.3.2 | PS calvin cyle rubisco small subunit |  |  |
| 9-cis-epoxycarotenoid dioxygenase (neoxanthin cleavage enzyme) | 184 |  | 17.1.1 | hormone metabolism abscisic acid synthesis-degradation |  |  |
| Peroxidase | 179 |  | 26.12 | misc peroxidases |  |  |
| Fruit protein oxidoreductase NAD binding domain-containing protein | 177 |  | 35.1 | not assigned no ontology |  |  |
| S-adenosyl-L-homocysteine hydrolase | 175 |  | 13.2.3.4 | amino acid metabolism degradation aspartate family methionine |  |  |
| Cinnamoyl CoA reductase | 174 |  | 16.2 | secondary metabolism phenylpropanoids |  |  |
| Inositol-3-phosphate synthase | 174 |  | 3.4.3 | minor CHO metabolism myo-inositol InsP Synthases |  |  |
| Aquaporin membrane intrinsic protein | 163 |  | 34.19.1 | transport Major Intrinsic Proteins PIP |  |  |
| Osmotin protein pathogenesis thaumatin family protein Alpha-amylase/trypsin inhibitor (Antifungal protein) | 161 |  | 20.1 | stress biotic |  |  |
| Actin related cluster | 157 |  | 31.1 | cell organisation |  |  |
| Polyvinylalcohol dehydrogenase | 154 |  | 35.2 | not assigned unknown |  |  |
| Hypothetical or unknown protein | 152 |  | 15 2 | metal handling binding, chelation and storage |  |  |
| 1-aminocyclopropane-1-carboxylate oxidase 1 (ACC oxidase 1) | 151 |  | 17 5 1 | hormone metabolism ethylene synthesis-degradation |  |  |
| Aromatic-L-amino-acid decarboxylase 2 | 144 |  | 16 4 1 | secondary metabolism N misc alkaloid-like |  |  |
| Major pollen allergen Bet V related (Bet V I-B) B I | 144 |  | 20 2 99 | stress abiotic unspecified |  |  |
| Alkaline alpha galactosidase | 140 |  | 3 1 2 2 | minor CHO metabolism raffinose family raffinose synthases putative |  |  |
| Raffinose alpha galactosidase seed imbibition protein | 138 |  | 3 1 2 2 | minor CHO metabolism raffinose family raffinose synthases putative |  |  |
| Major latex like protein | 137 |  | 35 2 | not assigned unknown |  |  |
| Pyruvate decarboxylase | 136 |  | 5 2 | fermentation PDC |  |  |
| Ripening protein; Barwin endoglucanase related cluster ; | 133 |  | 35 2 | not assigned unknown |  |  |
| Zinc finger homeobox family protein | 129 |  | 27 3 80 | RNA regulation of transcription zf-HD |  |  |
| Elongation factor 1-alpha (EF-1-alpha) | 126 |  | 29 2 4 | protein synthesis elongation |  |  |
| Expansin (EXP15) | 125 |  | 10 7 | cell wall modification |  |  |
| Endochitinase 3 | 124 |  | 20 1 | stress biotic |  |  |
| Hypothetical or unknown protein | 116 |  | 15 2 | metal handling binding, chelation and storage |  |  |
| Vacuolar ATP synthase 16 kDa | 112 |  | 34 1 | transport p- and v-ATPases |  |  |
| Harpin induced protein | 111 |  | 35 1 | not assigned no ontology |  |  |
| S-adenosylmethionine synthetase 2 | 107 |  | 15 2 | metal handling binding, chelation and storage | 13 1 3 4 | amino acid metabolism synthesis aspartate family methionine |
| Hypothetical or unknown protein | 106 |  | 35 2 | not assigned unknown |  |  |
| Chlorophyll a-b binding protein | 103 |  | 1 1 1 1 | PS light reaction photosystem II LHC-II |  |  |
| Cysteine proteinase | 103 |  | 29 5 3 | protein degradation cysteine protease |  |  |
| Thiazole biosynthetic enzyme | 103 |  | 35 1 | not assigned no ontology |  |  |

1. Thimm O, Blasing O, Gibon Y, Nagel A, Meyer S, Kruger P, Selbig J, Muller LA, Rhee SY, Stitt M**: MAPMAN: a user-driven tool to display genomics data sets onto diagrams of metabolic pathways and other biological process**es*. Plant* J 2004**,** 37(6):914-939.
