# Supplementary material for: Analysis of expressed sequence tags from Actinidia: applications of a cross species EST database for gene discovery in the areas of flavor, health, color and ripening
Source: BMC Genomics. 2008 Jul 27;9:351. doi: 10.1186/1471-2164-9-351 (PMC2515324; doi:10.1186/1471-2164-9-351)
Supplement: Additional file 6 — Additional Table 6. Most frequent InterPro families found in Actinidia NRs. [file 1471-2164-9-351-S6.doc]

Additional Table 6. Most frequent InterPro families found in *Actinidia* NRs.

| InterPro No. | NR Frequency^a^ | Description | Combined Frequency |
| --- | --- | --- | --- |
| IPR000719 | 758 | Protein kinase | 1535 |
| IPR011009 | 777 | Protein kinase-like |
| IPR013766 | 76 | Thioredoxin domain | 820 |
| IPR006663 | 101 | Thioredoxin domain 2 |
| IPR012335 | 274 | Thioredoxin fold |
| IPR012336 | 275 | Thioredoxin-like fold |
| IPR006662 | 94 | Thioredoxin-related |
| IPR002290 | 325 | Serine/threonine protein kinase | 571 |
| IPR008271 | 246 | Serine/threonine protein kinase, active site |
| IPR001356 | 61 | Homeobox | 460 |
| IPR009057 | 233 | Homeodomain-like |
| IPR012287 | 166 | Homeodomain-related |
| IPR013210 | 82 | Leucine rich repeat, N-terminal | 433 |
| IPR001611 | 209 | Leucine-rich repeat |
| IPR007090 | 142 | Leucine-rich repeat, plant specific |
| IPR001680 | 213 | WD40 repeat | 400 |
| IPR011046 | 187 | WD40-like |
| IPR013128 | 147 | Peptidase C1A, papain | 371 |
| IPR000668 | 127 | Peptidase C1A, papain C-terminal |
| IPR000169 | 97 | Peptidase, cysteine peptidase active site |
| IPR012677 | 351 | Nucleotide-binding, alpha-beta plait | 351 |
| IPR001245 | 336 | Tyrosine protein kinase | 336 |
| IPR000504 | 294 | RNA-binding region RNP-1 (RNA recognition motif) | 294 |
| IPR013026 | 85 | Tetratricopeptide region | 291 |
| IPR011990 | 206 | Tetratricopeptide-like helical |
| IPR007125 | 115 | Histone core | 273 |
| IPR009072 | 158 | Histone-fold |
| IPR001461 | 114 | Peptidase A1, pepsin | 255 |
| IPR009007 | 141 | Peptidase aspartic, catalytic |
| IPR001841 | 237 | Zinc finger, RING-type | 237 |
| IPR008994 | 129 | Nucleic acid-binding, OB-fold | 216 |
| IPR012340 | 87 | Nucleic acid-binding, OB-fold, subgroup |
| IPR008985 | 114 | Concanavalin A-like lectin/glucanase | 204 |
| IPR013320 | 90 | Concanavalin A-like lectin/glucanase, subgroup |
| IPR011989 | 181 | Armadillo-like helical | 181 |
| IPR000626 | 162 | Ubiquitin | 162 |
| IPR010987 | 90 | Glutathione S-transferase, C-terminal-like | 161 |
| IPR004045 | 71 | Glutathione S-transferase, N-terminal |
| IPR001806 | 154 | Ras GTPase | 154 |
| IPR001128 | 152 | Cytochrome P450 | 152 |
| IPR000008 | 68 | C2 | 145 |
| IPR008973 | 77 | C2 calcium/lipid-binding region, CaLB |
| IPR001005 | 143 | Myb, DNA-binding | 143 |
| IPR011992 | 138 | EF-Hand type | 138 |
| IPR011050 | 133 | Virulence factor, pectin lyase fold | 133 |
| IPR002110 | 131 | Ankyrin | 131 |
| IPR000608 | 131 | Ubiquitin-conjugating enzyme, E2 | 131 |
| IPR002048 | 130 | Calcium-binding EF-hand | 130 |
| IPR003579 | 130 | Ras small GTPase, Rab type | 130 |
| IPR013126 | 63 | Heat shock protein 70 | 125 |
| IPR001023 | 62 | Heat shock protein Hsp70 |
| IPR007124 | 124 | Histone-fold/TFIID-TAF/NF-Y | 124 |
| IPR000425 | 122 | Major intrinsic protein | 122 |
| IPR001810 | 121 | Cyclin-like F-box | 121 |
| IPR000757 | 62 | Glycoside hydrolase, family 16 | 120 |
| IPR000490 | 58 | Glycoside hydrolase, family 17 |
| IPR003612 | 119 | Plant lipid transfer/seed storage/trypsin-alpha amylase inhibitor | 119 |
| IPR013753 | 117 | Ras | 117 |
| IPR008972 | 115 | Cupredoxin | 115 |
| IPR005225 | 111 | Small GTP-binding protein domain | 111 |
| IPR001623 | 108 | Heat shock protein DNAJ, N-terminal | 108 |
| IPR012337 | 108 | Polynucleotidyl transferase, Ribonuclease H fold | 108 |
| IPR000379 | 106 | Esterase/lipase/thioesterase | 106 |
| IPR001509 | 103 | NAD-dependent epimerase/dehydratase | 103 |
| IPR002213 | 103 | UDP-glucuronosyl/UDP-glucosyltransferase | 103 |
| IPR001993 | 101 | Mitochondrial substrate carrier | 101 |

^a^  The list shows Interpro families with more than 100 *Actinidia* NR members when related families were summed together.
