# Supplementary material for: Analysis of expressed sequence tags from Actinidia: applications of a cross species EST database for gene discovery in the areas of flavor, health, color and ripening
Source: BMC Genomics. 2008 Jul 27;9:351. doi: 10.1186/1471-2164-9-351 (PMC2515324; doi:10.1186/1471-2164-9-351)
Supplement: Additional file 7 — Additional Table 7. Volatiles, acids, and sugars in Actinidia flowers and fruit [file 1471-2164-9-351-S7.doc]

Additional Table 7. Volatiles, acids, and sugars in *Actinidia* flowers and fruit. Four different analytical methods were used. For volatile compounds (esters, alcohols, fatty acids and terpenes), headspace extract values are given in brackets alongside solvent extract values. The volatile content is expressed as a percentage of the total compounds measured by that method. Where the percentages do not add up to 100%, other classes of compounds such as ketones, aldehydes and lactones that have not been included. In addition, unknown compounds that were also identified are also not listed. For acids, and sugars, the compound contents are expressed in mg/g. An empty cell indicates the compound was not detected and in the shaded cells, no analysis was performed.

|  | **Flower** | | | | | | **Fruit** | | | | |
| --- | --- | --- | --- | --- | --- | --- | --- | --- | --- | --- | --- |
| **Component** | *A. arguta* ‘Hortgem Tahi’ | *A. chinensis*(a) | *A. deliciosa* ‘Hayward’ | *A. eriantha* | *A. indochinensis* | *A. polygama* | *A. arguta* ‘Hortgem Tahi’ | *A. chinensis* ‘Hort 16A’ | *A. chinensis*  (a) | *A. deliciosa* | *A. eriantha* |
| **Esters** |  |  |  |  |  |  |  |  |  |  |  |
| 1-methylethyl tetradecanoate |  |  |  |  |  |  | (0.1) 4.0 |  |  | (0.02) |  |
| 2-phenylethyl acetate |  |  | (1.6) |  |  |  |  |  |  |  |  |
| 2-phenylethyl butanoate |  |  | (0.1) |  |  |  |  |  |  |  |  |
| 2-phenylethyl phenylacetate |  |  | 0.8 |  |  |  |  |  |  |  |  |
| 2-phenylethyl benzoate |  | 0.3 |  |  |  |  |  |  |  |  |  |
| 3-hydroxybutyl benzoate |  |  |  |  |  |  |  | 0.2 |  |  |  |
| benzyl benzoate |  |  |  |  |  |  | 0.7 | 0.03 |  |  |  |
| butyl 3-hydroxybutanoate |  |  |  |  |  |  |  |  | 0.2 |  |  |
| butyl acetate |  |  |  |  |  |  |  | (0.01) | (0.1) 0.1 | (0.07) | 12 |
| butyl benzoate |  |  |  |  |  |  |  | (0.01) 0.3 |  |  |  |
| butyl but-2-enoate |  |  |  |  |  |  |  | (0.01) | 0.2 |  |  |
| butyl butanoate |  |  |  | (1.2) |  |  |  | (0.1) 0.7 | (2.1) 21 | 1.0 |  |
| butyl hexanoate |  |  |  |  |  |  |  |  | 2.3 |  |  |
| ethyl 3-hydroxybutanoate |  |  |  |  |  |  |  |  | 0.1 |  |  |
| ethyl acetate |  |  | (0.3) |  |  |  | (0.4) | (0.1) 0.5 | (0.5) | (16) 1.4 | (0.5) |
| ethyl benzoate |  |  |  |  |  |  | (0.04) 5.4 | (0.03) 0.1 | (0.1) 0.4 |  |  |
| ethyl but-*E2*-enoate |  |  |  |  |  |  | 0.1 |  | (0.1) 0.2 |  |  |
| ethyl butanoate |  |  |  |  |  |  | (2.2) 7.6 | (3.1) 18 | (54) 53 | (11) 56 |  |
| ethyl decanoate |  |  |  |  |  |  |  | 0.2 |  |  |  |
| ethyl hex-2-enoate |  |  |  |  |  |  |  | (0.02) |  |  |  |
| ethyl hex-*E3*-enoate |  |  |  |  |  |  |  | (0.02) |  |  |  |
| ethyl hexadec-9-enoate |  |  |  |  |  |  | 3.5 |  |  |  |  |
| ethyl hexadecanoate |  |  |  |  |  |  | 4.9 |  |  |  | 1.2 |
| ethyl hexanoate |  |  |  |  |  |  | 2.4 | 1.2 | (0.9) 3.6 | (0.03) 0.9 |  |
| ethyl linoleate |  |  |  |  |  |  | 5.6 |  |  |  |  |
| ethyl linolenate |  |  |  |  |  |  | 8 |  |  |  |  |
| ethyl octanoate |  |  |  |  |  |  | 3.8 | (0.03) 0.3 | 0.3 |  |  |
| ethyl oleate |  |  |  |  |  |  | 1.1 |  |  |  |  |
| ethyl pentanoate |  |  |  |  |  |  | 0.1 | (0.01) 0.1 | (0.1) 0.2 |  |  |
| ethyl propanoate |  |  |  |  |  |  |  | (0.04) | (0.5) | (0.24) |  |
| heneicosyl formate |  |  |  |  |  | 1 |  |  |  |  |  |
| heptyl acetate |  |  |  | (2.2) |  |  | 0.2 |  |  |  |  |
| hex-*E2*-enyl acetate |  |  | (0.1) |  |  |  |  |  |  |  |  |
| hex-*E2*-enyl butanoate |  |  |  |  |  |  |  | 0.1 |  |  |  |
| hex-*E3*-enyl 2-methylbutanoate |  |  | (0.1) |  |  |  |  |  |  |  |  |
| hex-*E3*-enyl butanoate |  |  | (1.4) |  |  |  |  |  |  |  |  |
| hex-*Z3*-enyl acetate |  |  | (7.9) |  |  |  |  |  |  |  |  |
| hex-*Z4*-enyl acetate |  |  | (0.03) |  |  |  |  |  |  |  |  |
| hexyl 2-methylbutanoate |  |  |  | (0.6) |  |  |  |  |  |  |  |
| hexyl acetate |  |  | (2) | (1.7) |  |  |  |  |  |  |  |
| hexyl benzoate |  |  |  |  |  |  |  | 0.2 |  |  |  |
| hexyl butanoate |  |  | (0.1) |  |  |  |  |  | 2.6 |  |  |
| hexyl hexanoate |  |  |  |  |  |  |  |  | 0.1 |  |  |
| methyl 2-furoate |  |  |  |  |  |  |  |  |  | 0.8 |  |
| methyl acetate |  |  | (0.1) |  |  |  |  | (0.3) |  | (1.3) |  |
| methyl benzoate |  |  |  |  |  |  | 2.3 | (0.02) 0.3 | (0.2) 1.9 | (0.04) 0.53 |  |
| methyl but-*E2*-enoate |  |  |  |  |  |  |  | (0.03) |  | (0.04) |  |
| methyl butanoate |  |  |  |  |  |  |  | (0.1) 0.3 | (18.2) 9.3 | (29) |  |
| methyl decanoate |  |  |  |  |  |  |  | 0.02 |  |  |  |
| methyl hexadecanoate |  |  |  |  |  |  | 8.4 |  |  |  |  |
| methyl hexanoate |  |  |  |  |  |  |  | (0.01) 0.1 | (0.5) 1.2 | (0.1) 3.6 |  |
| methyl linoleate |  |  |  |  |  |  | 6.7 |  |  |  |  |
| methyl linolenate |  |  |  |  |  |  | 0.6 |  |  |  |  |
| methyl octadecanoate |  |  |  |  | 0.9 |  | 0.9 |  |  |  |  |
| methyl oleate |  |  |  |  | 21 |  | 4 |  |  |  |  |
| methyl pentanoate |  |  |  |  |  |  |  |  | (0.1) |  |  |
| methyl prop-2-enoate |  |  |  |  |  |  |  |  |  |  |  |
| methyl propanoate |  |  |  |  |  |  |  |  | (0.2) | (0.2) |  |
| methyl salicylate |  |  |  |  |  |  |  | 0.2 |  |  |  |
| pentyl acetate |  |  | (0.04) |  |  |  |  |  |  |  |  |
| pentyl butanoate |  |  |  |  |  |  |  |  | 0.1 |  |  |
| pent-*Z2*-enyl acetate |  |  | (0.3) |  |  |  |  |  |  |  |  |
| propyl butanoate |  |  |  |  |  |  |  | (0.02) 0.03 | (0.2) 0.4 |  |  |
|  |  |  |  |  |  |  |  |  |  |  |  |
| **Alcohols** |  |  |  |  |  |  |  |  |  |  |  |
| 1-methoxypropan-2-ol | (2.9) |  | (0.03) |  |  |  |  |  |  |  |  |
| 2-butoxyethanol |  |  | (0.03) |  |  |  |  |  |  |  |  |
| 2-ethylhexanol |  |  |  |  |  |  |  | 0.1 |  |  |  |
| 2-methylbut-3-en-2-ol | (0.8) |  |  |  | (0.5) |  |  |  |  |  |  |
| 2-methylbutanol | (12) |  |  |  |  | 0.01 |  |  |  |  |  |
| 2-methylpropanol | (0.1) |  |  |  | (0.1) | 0.01 |  |  |  |  | 1.4 |
| 2-(4-hydroxyphenyl)ethanol | 11 |  |  |  |  |  |  |  |  |  |  |
| 2-(4-methoxyphenyl)ethanol | 31 |  |  |  |  |  |  |  |  |  |  |
| 2-phenylethanol | (14) 8.6 | 5.8 | (7.9) 6.7 | (8.5) | (26) |  |  |  |  |  |  |
| 3-methylbutanol |  |  | (0.8) |  | (1.4) |  | 0.1 |  |  |  |  |
| 3-methylpentanol |  |  |  |  |  | 0.04 |  |  |  |  |  |
| benzyl alcohol |  |  |  |  | (2.6) |  | 0.7 |  |  |  |  |
| butanol |  |  | (0.1) | (1.3) | (0.3) |  |  | (0.2) 0.3 | (0.2) 0.1 | (0.15) |  |
| decanol |  |  |  |  |  |  | 0.7 |  |  |  |  |
| dodecanol |  |  |  |  |  |  | 7.8 |  |  |  |  |
| ethanol | (1) |  | (2) |  | (4.5) |  | (11.1) | (84) | (9.9) | (16.5) | (15) |
| heptanol |  |  |  |  |  |  |  | (0.1) |  |  |  |
| hex-*E2*-enol |  |  | (0.05) |  |  | 0.3 | 0.1 | (0.3) 2.4 | (0.6) 0.2 | 1.5 | (2.7) |
| hex-*E3*-enol |  |  | (0.1) |  |  |  |  | (0.005) 0.1 |  | (0.02) |  |
| hex-*Z3*-enol | (0.3) | 1.2 | (4.4) |  | (2.3) | 0.1 | 0.1 |  |  |  |  |
| hex-*Z4*-enol |  |  |  |  |  |  |  |  |  |  | (0.9) |
| hexadecanol | 0.5 |  | (2.8) |  |  |  | 0.8 |  |  |  |  |
| hexanol | (0.2) |  |  |  | (0.3) | 1 | 0.2 | (0.1) 2 | (0.3) 0.2 | (0.5) | (0.6) |
| methanol | (1) |  | (0.2) |  | (0.3) |  |  | (0.4) | (0.2) | (0.16) | (0.5) |
| nonanol |  |  |  |  |  |  | 0.2 | (0.005) |  |  |  |
| octanol |  |  | (0.1) |  |  |  | 1 | 0.03 |  |  |  |
| pentan-2-ol |  |  |  |  |  |  |  | 0.7 |  | (0.02) | 2.5 |
| pentanol |  |  | (0.1) |  |  |  |  | (0.04) 0.5 | 0.1 | (0.004) | 3.3 |
| penten-3-ol |  |  | (0.2) |  | (0.4) |  | (0.1) | (0.06) 0.04 |  | (0.09) |  |
| propanol |  |  | (0.02) |  |  |  |  |  |  |  |  |
|  |  |  |  |  |  |  |  |  |  |  |  |
| **Fatty Acids** |  |  |  |  |  |  |  |  |  |  |  |
| 2-ethylhexanoic acid |  |  |  |  |  |  |  | 1.5 |  |  |  |
| acetic acid | (3.9) |  | (0.3) |  | (1.5) | 0.1 |  | (0.8) 0.3 | (0.1) | (0.14) | (2.5) |
| butanoic acid |  |  |  |  |  |  |  | 0.2 | 0.1 |  |  |
| decanoic acid |  |  |  |  |  |  |  | 0.4 |  |  |  |
| hexadecanoic acid |  |  |  |  |  |  |  | 0.05 |  |  |  |
| hexanoic acid |  |  |  |  |  |  |  | 1.8 | 0.1 |  |  |
| hex-*E2*-enoic acid |  |  |  |  |  |  |  | 0.5 |  |  |  |
| nonanoic acid |  |  |  |  |  |  |  | 0.4 |  |  |  |
| octanoic acid |  |  |  |  |  |  |  | 10 |  |  |  |
| pentanoic acid |  |  |  |  |  |  |  | 0.1 |  |  |  |
| tetradecanoic acid |  |  |  |  |  |  |  | 0.03 |  |  |  |
|  |  |  |  |  |  |  |  |  |  |  |  |
| **Terpenes** |  |  |  |  |  |  |  |  |  |  |  |
| 6-methylhept-5-en-2-one | (0.1) |  | (0.1) | (8.5) | (0.1) | 0.01 | 0.1 | (0.03) 0.1 |  | (0.02) |  |
| camphor |  |  |  |  |  |  | 7.3 |  |  |  |  |
| car-2-en-4-ol |  |  |  |  |  |  |  | 0.5 |  |  |  |
| carene, 2- |  |  |  |  |  |  | 0.2 |  |  |  |  |
| carveol, cis- |  |  |  |  |  |  | 0.2 |  |  |  |  |
| carvone |  |  |  |  |  |  | 0.3 |  |  |  |  |
| caryophyllene, β- | (0.8) |  |  |  |  |  |  |  |  |  |  |
| cineole, 1,8-^a^ | (0.3) |  |  |  |  |  | 0.4 | (0.2) 33 | (0.1) 0.1 |  |  |
| cineole, exo-2-hydroxy- |  |  |  |  |  |  |  | 0.2 |  |  |  |
| cineole, 3-hydroxy-^b^ |  |  |  |  |  |  |  | 0.1 |  |  |  |
| cineole, 3-oxo- |  |  |  |  |  |  |  | 0.9 |  |  |  |
| cinnamyl alcohol |  |  |  |  |  |  |  | 0.02 |  |  |  |
| citronellol, β- |  |  | (5.4) |  |  | 4.5 |  |  |  |  |  |
| cymene, *p-* |  |  |  |  |  |  |  | (0.1) 0.4 |  |  |  |
| dehydroiridodial |  |  |  |  |  | 37 |  |  |  |  |  |
| elemene, δ- |  |  | (0.01) |  |  |  |  |  |  |  |  |
| elemene, γ- |  |  | (0.1) |  |  |  |  |  |  |  |  |
| elemol |  |  | 1.7 |  |  |  |  |  |  |  |  |
| farnesene, α- |  |  | (26.2) 12 |  |  |  |  |  |  |  |  |
| farnesol, 2,3-dihydro- |  | 23 |  |  |  | 7.4 |  |  |  |  |  |
| farnesol, *E,E*- |  | 44 |  |  |  |  |  |  |  |  |  |
| farnesylacetone, hexahydro- |  | 3.4 |  |  | 0.4 |  |  |  |  |  |  |
| geraniol, *E*- |  |  | (0.03) |  |  |  |  |  |  |  |  |
| geranylacetone | (0.4) |  | (0.02) |  |  |  |  | (0.03) 0.2 |  | (0.04) |  |
| germacrene B |  |  | (0.2) |  |  |  |  |  |  |  |  |
| germacrene D | (1.3) |  | (12) 0.3 |  |  |  |  |  |  |  |  |
| 6,7-dehydro-7,8-dihydro-3-oxo-α-ionol (isomer 1) |  |  |  |  | 10 |  |  |  |  |  |  |
| 6,7-dehydro-7,8-dihydro-3-oxo-α-ionol (isomer 2) |  |  |  |  | 1.6 |  |  |  |  |  |  |
| ionone, *E*-β- |  | 5 |  |  |  |  |  |  |  |  |  |
| kaur-16-ene |  |  |  |  | 6.7 | 3.7 |  |  |  |  |  |
| lilac alcohol a | (12) 8.3 |  |  |  |  |  |  |  |  |  |  |
| lilac alcohol b | 1 |  |  |  |  |  |  |  |  |  |  |
| lilac alcohol c | (1) 1.2 |  |  |  |  | 0.3 |  |  |  |  |  |
| lilac alcohol d | (3.8) 0.5 |  |  |  |  |  |  |  |  |  |  |
| lilac alcohol epoxide (2) | 0.2 |  |  |  |  |  |  |  |  |  |  |
| lilac alcohol epoxide (5) | 1.7 |  |  |  |  |  |  |  |  |  |  |
| lilac alcohol epoxide (6) | 0.1 |  |  |  |  |  |  |  |  |  |  |
| lilac alcohol epoxide (8) | 0.1 |  |  |  |  |  |  |  |  |  |  |
| lilac aldehyde 1 | (1.9) 0.1 |  |  |  |  |  |  |  |  |  |  |
| lilac aldehyde 2 | (0.5) |  |  |  |  |  |  |  |  |  |  |
| lilac aldehyde 3 | (0.8) 0.1 |  |  |  |  |  |  |  |  |  |  |
| lilac aldehyde 4 | (0.4) |  |  |  |  |  |  |  |  |  |  |
| limonene |  |  | (0.03) |  |  |  | 0.02 | 0.02 |  |  |  |
| linalool | (0.4) |  | (0.9) 0.0005 |  | (24) | 9.5 | 0.3 |  |  |  |  |
| epoxylinalol, *trans*- (pyranoid) |  |  |  |  |  | 0.9 |  |  |  |  |  |
| linalool oxide, *cis*- (furanoid) |  |  |  |  |  | 2.1 |  |  |  |  |  |
| linalool oxide, *trans*- (furanoid) |  |  |  |  |  | 0.3 |  |  |  |  |  |
| linalool, 5,6-dehydro- |  |  |  |  |  | 0.4 |  |  |  |  |  |
| linalool, 5-hydroxy- |  |  |  |  |  | 23 |  |  |  |  |  |
| linalool, 8-hydroxy- (*Z*-isomer) |  |  |  |  | 1.6 |  |  |  |  |  |  |
| linalool, 8-hydroxy- (*E*-isomer) | 0.2 |  |  |  |  |  |  |  |  |  |  |
| octa-3,7-diene-2,6-diol,  2,6-dimethyl- |  |  |  |  |  | 0.7 |  |  |  |  |  |
| menthen-4-ol, *p*- |  |  |  |  |  |  |  | 0.4 |  | 0.06 |  |
| menthene, p- |  |  |  |  |  |  |  | (0.02) |  |  |  |
| menthol |  |  |  |  |  |  | 0.8 |  |  |  |  |
| myrcene, β- |  |  | (0.02) | (5) |  |  |  | 0.1 |  |  |  |
| nerolidol, *E*- |  | 0.3 |  |  |  |  |  |  |  |  |  |
| norbornan-2-one, endo-5,5,6-trimethyl- |  |  |  |  |  |  | 0.4 |  |  |  |  |
| ocimene, β- | (0.1) |  | (1.3) |  | (0.8) |  |  |  |  |  |  |
| phytol |  | 4.7 |  |  | 4.4 |  |  |  |  |  |  |
| pinan-3-one |  |  |  |  |  |  |  | 0.3 |  |  |  |
| pinene, α- | (0.3) |  | (0.02) |  |  |  | (0.1) 0.1 | 0.2 |  |  | (1.6) |
| pinene, β- | (0.1) |  |  |  |  |  | (0.7) | 0.2 | (0.1) |  | (2.2) |
| piperitone |  |  |  |  |  |  |  | 0.2 |  |  |  |
| sabinene | (0.6) |  |  |  |  |  |  |  |  |  |  |
| squalene | 12 |  |  |  | 0.6 |  | 0.2 |  |  |  | 13 |
| terpinene, γ- |  |  |  |  |  |  |  | 0.1 |  |  |  |
| terpineol, α- |  |  |  |  |  |  | 1.2 |  |  |  |  |
| terpinolene |  |  |  |  |  |  | (0.1) | 0.01 |  |  | (1.9) |
|  |  |  |  |  |  |  |  |  |  |  |  |
| **Acids (mg/g)** |  |  |  |  |  |  |  |  |  |  |  |
| ascorbic |  | 2.1 | 0.62 | 4.8 |  |  |  |  | 1.5 |  | 10 |
| citric acid |  |  | 0.3 | 0.3 |  | 0.1 | 5.6 |  | 8.8 | 9.5 | 14 |
| malic acid |  |  | 2.0 | 2.1 |  | 1.5 | 2.6 |  | 5.3 | 3.8 | 4.2 |
| quinic acid |  |  | 1.3 | 0.1 |  |  | 5.2 |  | 9.5 | 9.9 | 4.3 |
|  |  |  |  |  |  |  |  |  |  |  |  |
| **Sugars (mg/g)** |  |  |  |  |  |  |  |  |  |  |  |
| fructose |  |  | 9.2 | 13 |  | 11 | 22 |  | 32 | 35 | 16 |
| galactose |  |  |  |  |  |  | 0.9 |  |  | 0.7 | 0.3 |
| glucose |  |  | 9.6 | 7.6 |  | 2.2 | 20 |  | 30 | 32 | 15 |
| *myo*-inositol |  |  | 0.75 | 0.9 |  | 0.53 | 3.3 |  | 1 | 1.1 | 0.5 |
| sucrose |  |  | 1.4 | 3.8 |  | 1.6 | 45 |  | 48 | 40 | 9.5 |

^a^eucalyptol; ^b^1,5,5-trimethyl-6-oxa-bicyclo[2.2.2]octan-3-ol
